# ROP39 is an Irgb10-specific parasite effector that modulates acute *Toxoplasma gondii* virulence

**DOI:** 10.1371/journal.ppat.1011003

**Published:** 2023-01-05

**Authors:** Shishir Singh, Mateo Murillo-León, Niklas Sebastian Endres, Ailan Farid Arenas Soto, Jorge Enrique Gómez-Marín, Florence Melbert, Thirumala-Devi Kanneganti, Masahiro Yamamoto, Claudia Campos, Jonathan Charles Howard, Gregory Alan Taylor, Tobias Steinfeldt

**Affiliations:** 1 Institute of Virology, Medical Center University of Freiburg, Freiburg, Germany; 2 Faculty of Medicine, University of Freiburg, Freiburg, Germany; 3 Faculty of Biology, University of Freiburg, Freiburg, Germany; 4 Grupo GEPAMOL, Centro de Investigaciones Biomedicas, Universidad del Quindio, Armenia, Quindio, Colombia; 5 Department of Immunology, St. Jude Children´s Research Hospital, Memphis, Tenessee, United States of America; 6 Department of Immunoparasitology, Research Institute for Microbial Diseases, Osaka University, Suita, Osaka, Japan; 7 Laboratory of Immunoparasitology, World Premier International Immunology Frontier Research Center, Osaka University, Suita, Osaka, Japan; 8 Fundacao Calouste Gulbekian, Instituto Gulbekian de Ciencia, Oeiras, Portugal; 9 Departments of Medicine; Molecular Genetics and Microbiology; and Immunology; and Center for the Study of Aging and Human Development, Duke University Medical Center, Durham, North Carolina, United States of America; 10 Geriatric Research, Education, and Clinical Center, Durham VA Health Care System, Durham, North Carolina, United States of America; Universitat Bern, SWITZERLAND

## Abstract

*Toxoplasma gondii* (*T*. *gondii*) is a zoonotic apicomplexan parasite that is an important cause of clinical disability in humans. On a global scale, one third of the human population is infected with *T*. *gondii*. Mice and other small rodents are believed to be responsible for transmission of *T*. *gondii* to the domestic cat, its definitive host. Interferon-inducible Immunity-Related GTPases (IRG proteins) are important for control of murine *T*. *gondii* infections. Virulence differences between *T*. *gondii* strains are linked to polymorphic rhoptry proteins (ROPs) that cooperate to inactivate individual IRG family members. In particular, the pseudokinase ROP5 isoform B is critically important in laboratory strains of mice. We identified *T*. *gondii* ROP39 in complex with ROP5B and demonstrate its contribution to acute *T*. *gondii* virulence. ROP39 directly targets Irgb10 and inhibits homodimer formation of the GTPase leading to an overall reduction of IRG protein loading onto the parasitophorous vacuolar membrane (PVM). Maintenance of PVM integrity rescues the parasite from IRG protein-mediated clearance *in vitro* and *in vivo*. This study identifies a novel *T*. *gondii* effector that is important for specific inactivation of the IRG resistance system. Our data reveal that yet unknown *T*. *gondii* effectors can emerge from identification of direct interaction partners of ROP5B.

## Author summary

Toxoplasmosis is one of the more common parasitic zoonoses world-wide. Its causative agent, *Toxoplasma gondii* (*T*. *gondii*), is an obligate intracellular food-borne parasite. It has a very broad host range establishing a productive infection in virtually all warm-blooded animals including humans. A mouse model of toxoplasmosis is of medical importance because the relationship with one of its most important natural hosts, the mouse, determines the impact of *T*. *gondii* infections in humans. In the acute phase of infection, the parasite seeks to reach an intermediate level of virulence to increase chances for transmission. Virulence is a multifactorial phenotype; we and others have described several effector proteins of the parasite that specifically inhibit certain host cell GTPases (IRG proteins). Only the combination of all parasite effectors determines the full virulence potential of the parasite. Here, we demonstrate that *T*. *gondii* has evolved a virulence effector, ROP39, that specifically targets Irgb10 to escape IRG-mediated control. This is a novel aspect of the infection biology of this world-wide zoonotic pathogen. It advances our understanding of the coevolutionary relationship of *T*. *gondii* and the mouse, an important intermediate host for transmission.

## Introduction

*Toxoplasma gondii* (*T*. *gondii*) is a zoonotic intracellular parasite that is capable of infecting almost all warm-blooded animals. The biphasic genetic population structure of *T*. *gondii* corresponds to a few highly clonal and abundant lineages in the Northern Hemisphere, whereas in other parts of the world, especially in South America, parasite genotypes are more diverse and appear to undergo more frequent recombination [1–3]. Analysis of three lineages that predominate in Europe and North America, referred to as clonal strains type I, II and III [4,5] demonstrated that type I strains are usually lethal at low inocula in laboratory strains of mice whereas type II and III strains are considered avirulent [6,7].

Most strains of *T*. *gondii* probably live in the wild in equilibrium with their principal natural hosts; they are not virulent and the early proliferative growth of tachyzoites is limited by effective resistance mechanisms that initiate the transition into slowly growing tissue cyst bradyzoites. The cost of infection to the resistant host is low (at least 30% of the human race are infected with *T*. *gondii*) and tissue cysts guarantee transmission by carnivorism. Virulence is a dysgenic property since early death of the host also eliminates the parasite. Nevertheless, *T*. *gondii* possesses a formidable armory of proven or potential virulence effectors which reveal themselves when, for example, a type I strain infects a laboratory mouse. It is, however, certain that *T*. *gondii* virulence effectors serve in nature not to kill potential hosts but to establish a long-term equilibrium against host-resistance.

Resistance against a wide range of vacuolar pathogens is mediated by two families of large GTPases, the Immunity-Related GTPases [8,9]. The Immunity-Related GTPases (IRG proteins) comprise a family of IFNγ-inducible proteins represented by about 20 single coding units in the C57BL/6 (BL/6) mouse genome [10–13]. BL/6 and many other laboratory mouse strains can be stably infected by *T*. *gondii* type II and III strains. However, this equilibrium breaks down in all such mouse strains when one or more *IRG* locus is deleted, where normally avirulent type II strains become lethal [14–17]. IRG protein accumulation at the *T*. *gondii* parasitophorous vacuolar membrane (PVM) is an indispensable resistance mechanism. It limits excessive growth and proliferation of the tachyzoite stage by disruption of enough vacuoles to rescue the host, and probably contributes to the transition of surviving tachyzoites to the slow-replicating tissue cyst bradyzoites [18–22]. Accumulation of certain IRG proteins and subsequent morphological vesiculation and disruption that can be observed at the microscopic level [18,12,22] indicates an effector function of these IRG proteins at the PVM. Effector IRG proteins that load onto the PVM upon infection are kept in a GDP-bound inactive conformation by IRG regulator proteins Irgm1, Irgm2 and Irgm3 at endogenous intracellular membranes in uninfected cells [23–26]. To preserve integrity of the PVM, *T*. *gondii* has evolved several polymorphic virulence effectors [27] that are injected from secretory organelles directly into the host cell cytosol.

Genetic screens initially revealed that single polymorphic rhoptry family members account for the differences in virulence between *T*. *gondii* strains [6,28–30]. The loading of the PVM with IRG proteins is markedly reduced in case of *T*. *gondii* type I compared with type II and III strains [31] resulting in virulence and early death of the host, and several effectors are described to inactivate these IRG proteins [32–40]. Phosphorylation of two conserved threonine residues in the switch I region of their nucleotide binding domain results in maintenance of PVM integrity allowing uncontrolled replication of the parasite. Whereas *T*. *gondii* ROP18 specifically phosphorylates Irga6 at the PVM [37,41], ROP17 could be demonstrated to preferentially phosphorylate Irgb6 [34]. Another virulence locus, shown to have the largest impact on strain-specific virulence [6,29], comprises a cluster of closely related polymorphic genes encoding the ROP5 pseudokinases. Three major isoforms, ROP5A, B and C, are encoded within the locus of each of the three clonal *T*. *gondii* strains [42]. Our previous results demonstrate a critical responsibility of ROP5B for virulence of *T*. *gondii* type I in laboratory strains of mice [43].

A reciprocal polymorphism of parasite effectors and IRG proteins presents strong evidence for a coevolutionary relationship of *T*. *gondii* and mice. Both genotypes determine the outcome of *T*. *gondii* infections [44]. In certain wild-derived mice, Irgb2-b1 directly binds and decoys ROP5B, allowing accumulation of IRG effector proteins like Irga6, Irgb6, Irgb10 and Irgd at the PVM and preventing uncontrolled parasite replication, enabling encystment and ultimately transmission. Laboratory strains of mice, however, express low levels of polymorphic Irgb2-b1 variants that cannot bind ROP5 [43]. We could demonstrate that ROP5 can directly block Irga6 function because ROP5 binding partially covers the Irga6 activation interface necessary for its oligomerisation *in vitro* [36]. In addition, association of ROP5 with ROP18 [37] confirms positive regulation of Irga6-specific ROP18 kinase activity [33]. ROP17 was also demonstrated to form a complex with ROP5 but, unlike ROP18, enzymatic activity of ROP17 *in vitro* seems to be independent from ROP5 [34]. The existence of ROP5-dependent, ROP18-independent virulence traits against IRG proteins has already been shown [33,36]. Furthermore, the double deletion mutant RHΔ*rop17*/*rop18* is avirulent even at high inocula but still not as compromised as *T*. *gondii* RHΔ*rop5* [34]. It therefore seems likely that ROP5 assists auxiliary *T*. *gondii* effectors for individual IRG protein inhibition.

In the present study, we screened several *T*. *gondii* rhoptry proteins for interaction with ROP5 isoform B to identify potential new effector proteins that contribute to acute virulence of the parasite. Our work identifies *T*. *gondii* ROP39 that is directly associated with ROP5B. We demonstrate that ROP39 is a novel effector of the parasite that specifically targets Irgb10. Inhibition of Irgb10 dimerisation results in overall reduction of IRG protein accumulation at the PVM. This ROP39-mediated vacuolar loading phenotype is reflected in attenuated parasite virulence *in vitro* and *in vivo*. Our data provide additional insight into the interplay between host cell resistance GTPases and *T*. *gondii* virulence effectors and reveal that novel effectors of the parasite can emerge from identification of interaction partners of ROP5.

## Results

### *T*. *gondii* ROP39 is directly associated with ROP5B

**Fig 1:**
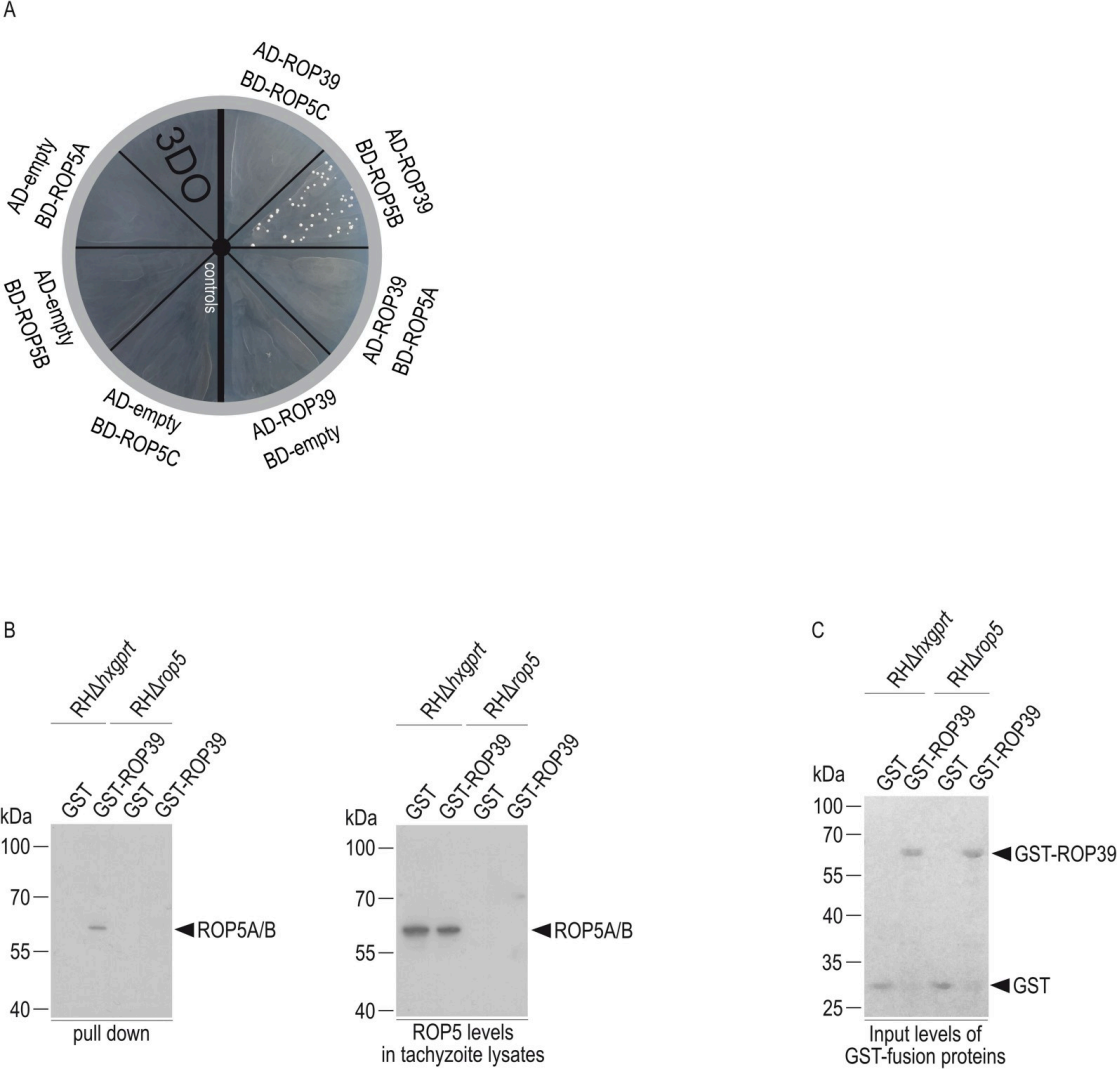
*T*. *gondii* ROP39 is directly associated with ROP5B. A, Yeast Two-Hybrid. *T*. *gondii* ROP39 directly binds to ROP5B but not ROP5A and ROP5C. Proteins were expressed either as fusion to the Gal4 transcriptional activation (AD) or DNA-binding (BD) domain. Colony growth under 3DO conditions demonstrates protein:protein-interaction. The bold black line indicates assembly of two different plates. B, Pull-down of ROP5A and/or ROP5B (the antibody used for detection does not allow detection of ROP5C and cannot discriminate between ROP5A and B [43]) by mature GST-ROP39 from RHΔ*hxgprt* tachyzoite detergent lysates. RHΔ*rop5* tachyzoite detergent lysates have been included as control (left hand panel). ROP5 levels in tachyzoite detergent lysates used in the pull-down (right hand panel). C, Ponceau staining of the membrane used in B shows the input of GST and GST-fusion proteins used in the pull-down.

The pseudokinase ROP5 plays a key role for *T*. *gondii* virulence and we demonstrated recently that ROP5 isoform B (ROP5B) is largely responsible [43]. In a Yeast Two-Hybrid (YTH) approach we screened several *T*. *gondii* rhoptry proteins (ROP11, ROP17, ROP20, ROP39) with predicted Ser/Thr kinase activity and PVM localization [45,46] for interaction with ROP5B. Colony growth on selective medium is indicative for a direct interaction after expression of two proteins as N-terminal fusions with the Gal4 DNA-binding (BD) or Gal4 activation domain (AD) in a yeast reporter strain. *T*. *gondii* RHΔ*hxgprt*-derived ROP39 was found to directly interact with ROP5B but not ROP5A or ROP5C (Fig 1A). ROP5B:ROP39-interaction was confirmed in pull-down experiments with a glutathione S-transferase (GST)-tagged ROP39 fusion protein on *T*. *gondii* detergent lysates and subsequent Western blot analysis using an anti-ROP5 antibody. (Fig 1B, left hand panel). Equal ROP5 amounts and validation of *T*. *gondii* strains were confirmed in detergent tachyzoite lysates (Fig 1B, right hand panel). Ponceau staining of the membrane before immunodetection indicates the input of GST and GST-ROP39 used in the pull-down (Fig 1C).

### ROP39 is proteolytically processed and localises to the PVM

**Fig 2:**
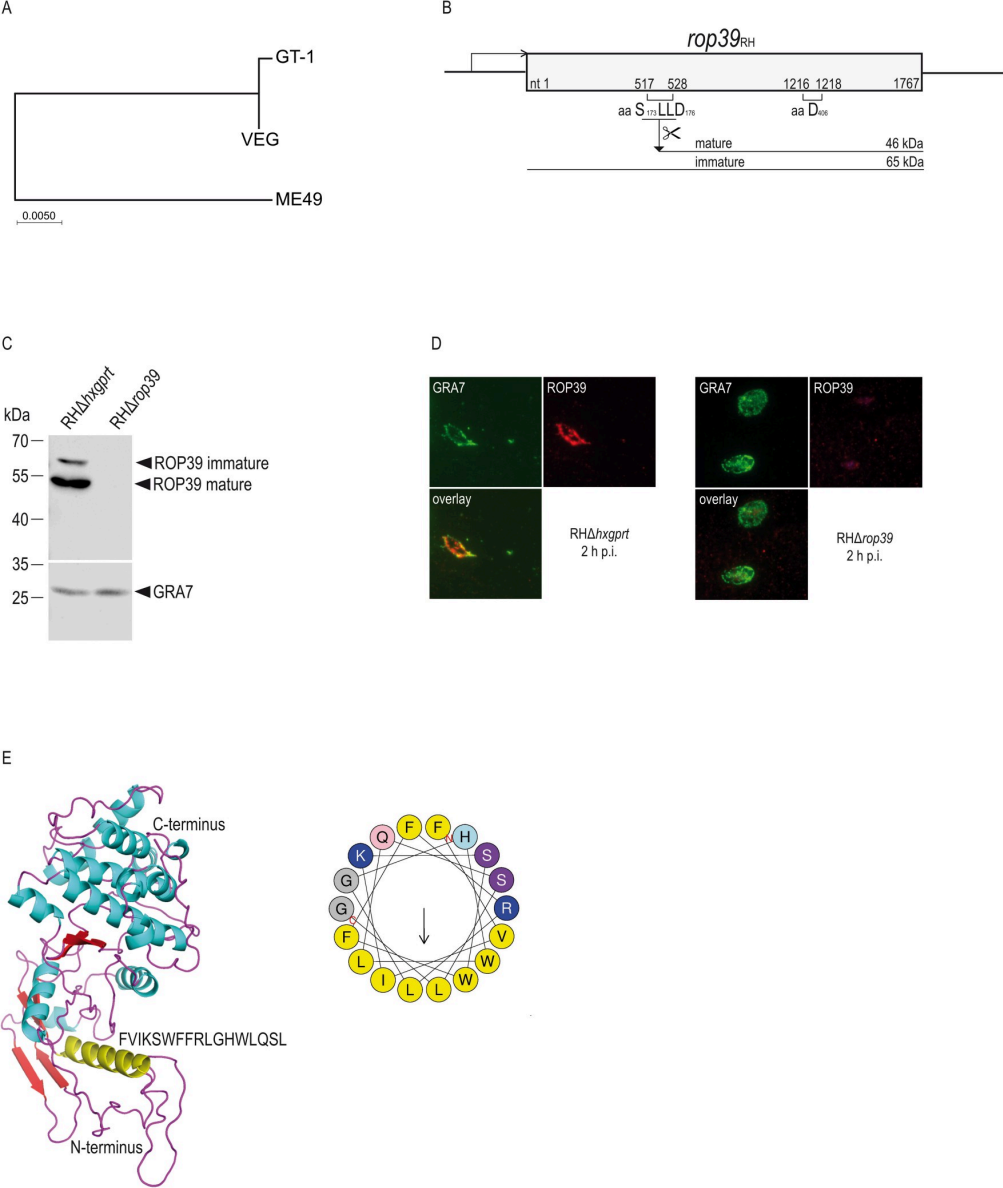
ROP39 is proteolytically processed and localises to the PVM. A, Phylogenetic analysis and maximum likelihood tree of *T*. *gondii* ROP39 amino acid sequences from indicated *T*. *gondii* strains. B, Schematic representation of *rop39*_RH_ at its endogenous locus. The sequence used for proteolytical cleavage (SLLD) of the immature protein and one key catalytic aspartate (D) is depicted. C, Generation of mature ROP39 after proteolytic cleavage and deletion of *rop39* in RHΔ*hxgprt* parasites is demonstrated by Western blot using a ROP39-specific peptide antiserum. D, ROP39 localizes to the PVM in infected host cells. Mouse embryonic fibroblasts were infected with *T*. *gondii* RHΔ*hxgprt* or RHΔ*rop39* for 2 h and subsequently stained with a ROP39-specififc peptide antiserum. Intracellular *T*. *gondii* were identified by GRA7 staining. E, 3D schematic representation of the ROP39 structure. The N-terminal amphipathic α-helix is shown in yellow. Amphipathic character is indicated by helical wheel projection. (See S1 Fig for data related to A, S2, S11 and S14 Figs for data related to C and S3 Fig for data related to E).

Phylogenetic analysis of *T*. *gondii* strains type I, II and III (Fig 2A) revealed almost identical ROP39 alleles in type I and III (one amino acid exchange (P to L) at position 368) that differ in 29 amino acids from the type II allele (S1 Fig). Several rhoptry proteins described so far are proteolytically processed by the subtilisin-like serine proteinase TgSUB2 at the proposed consensus sequence SΦXE [47]. ROP39 contains the motif S_173_LLD_176_ (Fig 2B) and proteolytic cleavage was demonstrated by Western blot from tachyzoite lysates using a ROP39-specific peptide antiserum (Fig 2C). As a specificity control, we generated a *rop39* deletion mutant (RHΔ*rop39*), deletion in which was confirmed by western blotting of tachyzoite lysates (Fig 2C) and diagnostic PCR (S2 Fig). Complex formation with ROP5B (Fig 1) would suggest—in analogy to ROP18 and ROP17—an Immunity-Related GTPases (IRG) inhibitory function of ROP39. All parasite effectors identified to date that inactivate IRG proteins are redirected to the PVM. Similarly, we found ROP39 to localize to the PVM (Fig 2D) confirming recently published data [48]. PVM association of ROP2 family proteins, such as ROP18, is mediated by a series of 3 N-terminal amphipathic α-helices [49]. However, amino acid sequence alignment revealed that in ROP39 these helices are highly degenerate (S3 Fig). Instead, a single large α-helix (amino acid F_44_-L_60_) is present (Fig 2E, left hand panel; S3 Fig) with predicted amphipathic character when plotted on a helical wheel (Fig 2E, right hand panel). However, whether this predicted α-helix mediates PVM association of ROP39 still remains to be determined.

### ROP39 inhibits Irga6 loading onto the PVM but is not another Irga6 kinase

**Fig 3:**
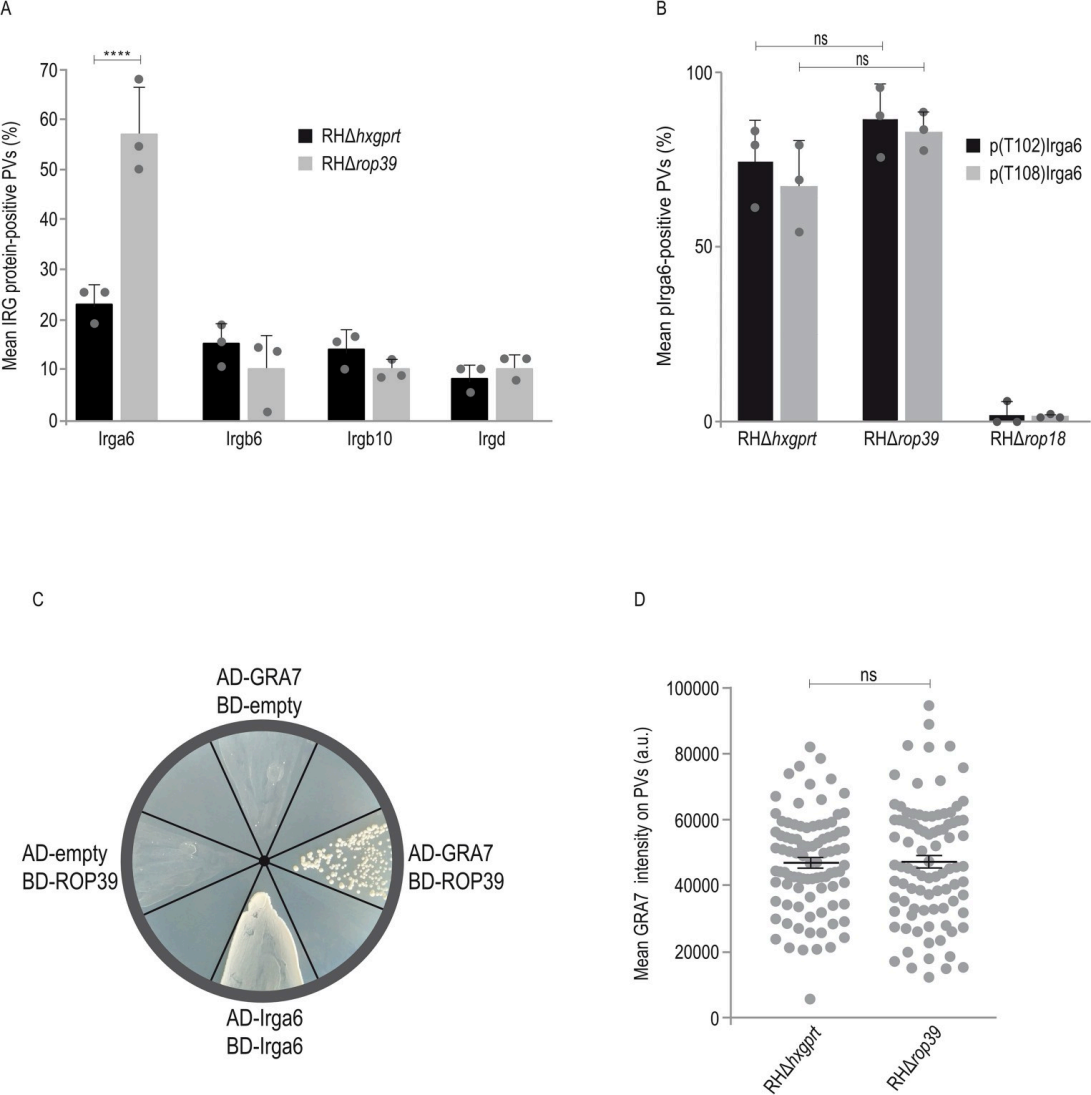
ROP39 inhibits Irga6 loading onto the PVM but is not another Irga6 kinase. A, B, Frequencies of vacuoles positive for Irga6, Irgb6, Irgb10 and Irgd (A) or Irga6 phosphorylated at T108 (p(T108)Irga6) or T102 (p(T102)Irga6) (B). Mouse embryonic fibroblasts were stimulated with IFNγ for 24 h and infected for 2 h before cells were prepared for immunofluorescence. Error bars indicate the mean and standard error of the mean (SEM) of three independent experiments (about 100 vacuoles were evaluated per experiment). Two-way analysis of variance (ANOVA) followed by Sidak´s multiple comparison was used to test differences between groups; ****p < 0.0001; ns not significant. A, Frequencies of Irga6-positive vacuoles were significantly increased in RHΔ*rop39* compared to RHΔ*hxgprt* infections. B, No differences in percentages of p(T108)Irga6- and p(T102)Irga6-positive vacuoles were detected in RHΔ*hxgprt* and RHΔ*rop39* infections whereas phosphorylation of Irga6 at T108 or T102 is almost completely absent in case of RHΔ*rop18*. C, Yeast Two-Hybrid analysis demonstrates direct binding of *T*. *gondii* ROP39 to GRA7. D, Intensities of GRA7 at the PVM determined by immunofluorescence with an anti GRA7 antibody. Mouse embryonic fibroblasts were stimulated with IFNγ for 24 h and infected for 2 h before cells were prepared for immunofluorescence. Error bars indicate the mean and standard error of the mean (SEM) of three independent experiments (about 30 vacuoles were evaluated in each experiment). Kruskal–Wallis test followed by Dunn’s multiple comparisons was used to test differences between groups; ns not significant. (See S2, S11 and S14 Figs for data related to A, B, D).

In previous studies, ROP5 was demonstrated to inhibit the IRG resistance system via direct association with Irga6 [36,38] and by enhancing ROP18 kinase activity [33]. To investigate whether complex formation with ROP5B reflects a possible impact of ROP39 on the IRG resistance system, we determined by immunofluorescence the frequencies of vacuoles positive for IRG effector proteins Irga6, Irgb6, Irgb10 and Irgd, 2 h post infection. Frequencies of vacuoles positive for Irga6 but not for any other IRG effector protein were increased in cells infected with RHΔ*rop39* relative to the parental strain RHΔ*hxgprt* (Fig 3A).

Inhibition of Irga6 is associated with ROP18-mediated phosphorylation of conserved threonine residues T102 and T108 in the switch I region of the GTPase domain [35,39]. When we immunoprecipitated Irga6 from cells metabolically labelled with ^33^P-phosphoric acid and infected with RHΔ*rop18*, no sign of phosphorylated Irga6 (pIrga6) could be detected in comparison to RH-YFP infected cells indicating that ROP18 is the only Irga6-specific *T*. *gondii* kinase [39]. To definitely exclude that T102 or T108 of Irga6 are targets of phosphorylation by ROP39, we enumerated p(T102)Irga6- and p(T108)Irga6-positive PVs using two phosphospecific antisera 2 h post infection of MEFs that had been stimulated for 24 h with IFNγ. No differences in frequencies of pIrga6-positive vacuoles were found in RHΔ*rop39* relative to parental strain infections (Fig 3B). The overall amount of p(T102)Irga6 and p(T108)Irga6 was determined by Western blot from RHΔ*rop39* and wt strain infected cells. No differences in p(T102)Irga6 or p(T108)Irga6 could be detected (S4 Fig). These results indicate that ROP39 is neither another Irga6 kinase nor does it contribute to Irga6-specific kinase activity of ROP18.

GRA7 is another *T*. *gondii* effector that is associated in a complex with ROP5 and ROP18 and contributes to Irga6 inhibition by enhancing ROP18 kinase activity [37]. We could detect direct binding of GRA7 to ROP39 by YTH analysis (Fig 3C). This finding implies that the Irga6 loading phenotype in absence of ROP39 (Fig 3A) is due to alteration of vacuolar GRA7 amounts. However, absence of ROP39 does not influence vacuolar GRA7 accumulation (Fig 3D). Therefore, presence of GRA7 within the ROP39/ROP5/GRA7 complex does not explain the inhibitory effect of ROP39 on Irga6.

### ROP39 contributes to virulence of *T*. *gondii* type I *in vivo*

**Fig 4:**
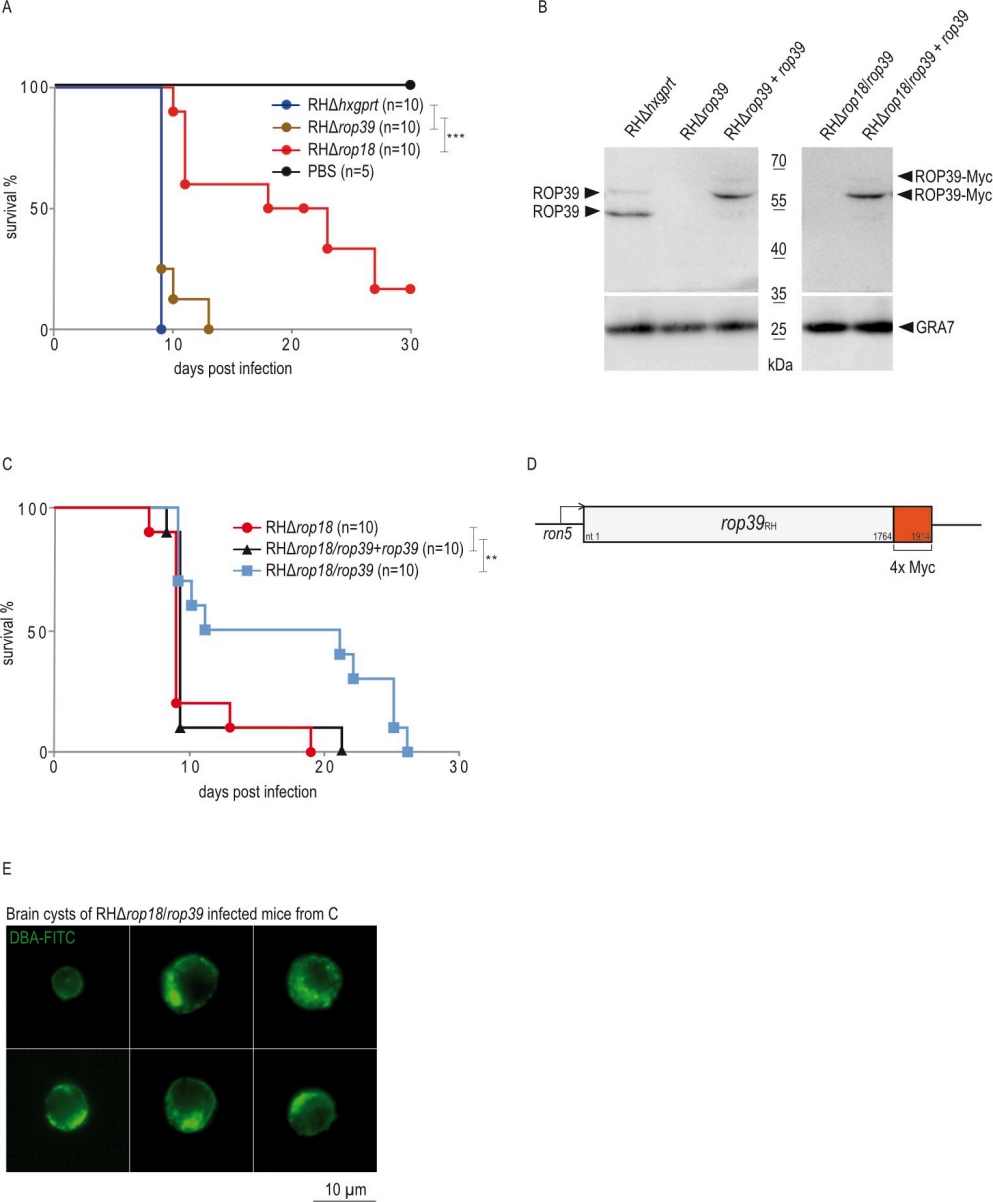
ROP39 contributes to virulence of *T*. *gondii* type I *in vivo*. A, Survival of C57BL/6 mice that were intraperitoneally infected with 200 freshly prepared tachyzoites of indicated *T*. *gondii* strains. A log-rank Mantel-Cox test was used to test differences between groups; ***p < 0.001. B, Deletion of *rop39* in RHΔ*rop18* parasites (RHΔ*rop18*/*rop39*) and complementation of ko strains with Myc-tagged *rop39* (RHΔ*rop39*+*rop39* and RHΔ*rop18*/*rop39*+*rop39*) is demonstrated in comparison to RHΔ*hxgprt* and RHΔ*rop39* by Western blot using a ROP39-specific peptide antiserum. C, Survival of C57BL/6 mice that were intraperitoneally infected with 1,000 freshly prepared tachyzoites of indicated *T*. *gondii* strains. A log-rank Mantel-Cox test was used to test differences between groups; **p < 0.01. D, Schematic representation of *T*. *gondii rop39*_RH_ under control of the *ron5* promoter. The Myc tag (4 x Myc) at the C-terminus is depicted in red. E, Brain homogenates from RHΔ*rop18*/*rop39* infected C57BL/6 mice were prepared 21, 22 and 25 days p.i. respectively and cysts stained with DBA-FITC. (See S2 and S14 Figs for data related to A, B, C, E, S6 Fig for data related to B, C and S11 Fig for data related to A, B).

Interaction with ROP5B (Fig 1) and disturbance of vacuolar Irga6 loading (Fig 3A) suggest that ROP39 is contributing to *T*. *gondii* virulence. To address this, we determined the impact of *rop39* on acute *T*. *gondii* virulence *in vivo* by inoculating C57BL/6 (BL/6) mice with 200 RHΔ*rop39* tachyzoites. Absence of ROP39 alone resulted in negligible attenuation of virulence relative to the parental control strain (Fig 4A). At present, two rhoptry kinases, ROP18 [32,34,36,37,39] and ROP39 (Fig 3A), are known to inhibit accumulation of Irga6 at the *T*. *gondii*-derived PVM. To investigate a possible redundancy of ROP39 and ROP18, we deleted *rop39* in a Δ*rop18* background and determined virulence in BL/6 mice in comparison with RHΔ*rop18*. Generation of the double ko *T*. *gondii* strain was confirmed by diagnostic PCR (S2 Fig) and Western blot (Fig 4B). In comparison to RHΔ*rop18*, virulence of the double deletion mutant RHΔ*rop18*/*rop39* is significantly attenuated at a dose of 1,000 tachyzoites (Fig 4C). Complementation of *rop39* in the double ko background (RHΔ*rop18*/*rop39*+*rop39*) with Myc-tagged ROP39 (Fig 4B and 4D) restored virulence to levels comparable with RHΔ*rop18* (Fig 4C). To permit propagation, *T*. *gondii* needs to form tissue cysts that serve as source of infection for intermediate and primary hosts. After examination of three RHΔ*rop18*/*rop39*-infected animals that had to be sacrificed due to severe weight loss (21, 22 and 25 days post infection), we detected tissue cysts in the brain demonstrating that a combined deletion of ROP18 and ROP39 induces attenuation of virulence that allows parasite transmission (Fig 4E).

### ROP39 is important to inactivate the IFNγ-inducible IRG response

**Fig 5:**
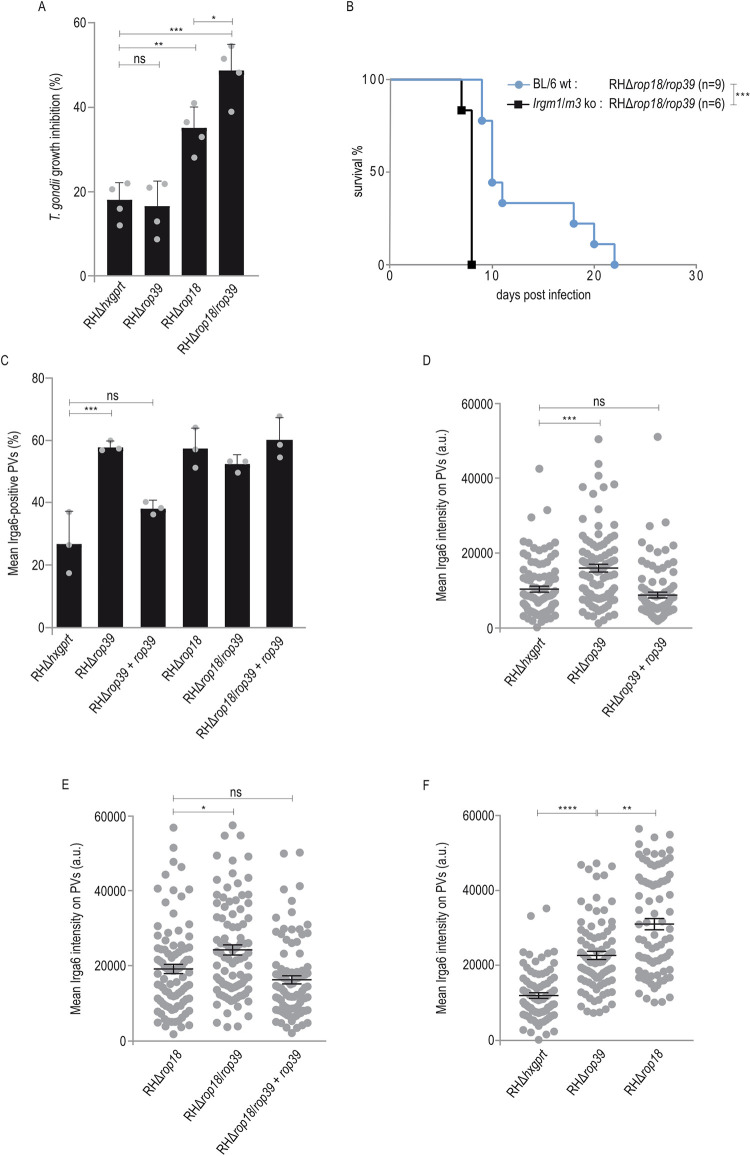
ROP39 is important to inactivate the IFNγ-inducible IRG response. A, Plaque assay. Mouse embryonic fibroblasts grown in 6 well-plates in presence or absence of IFNγ (100 U/ml) for 24 h were infected with 200 freshly prepared *T*. *gondii* tachyzoites. At 7 d post infection, cell monolayers were stained with crystal violet. Error bars indicate the mean and standard error of the mean (SEM) of four independent experiments. One-way analysis of variance (ANOVA) followed by Tukey´s multiple comparison was used to test differences between groups; ***p < 0.001; **p < 0.01; *p < 0.05; ns not significant. B, Survival of C57BL/6 *Irgm1*/*Irgm3* ko or wt mice that were intraperitoneally infected with 1,000 freshly prepared tachyzoites of *T*. *gondii* RHΔ*rop18*/*rop39*. A log-rank Mantel-Cox test was used to test differences between groups; ***p < 0.0002. Frequencies (C) and intensities (D, E, F) of Irga6-positive vacuoles. Mouse embryonic fibroblasts were stimulated with IFNγ for 24 h and infected for 2 h before cells were prepared for immunofluorescence and stained with an anti Irga6-specific antibody. Error bars indicate the mean and standard error of the mean (SEM) of three independent experiments. C, One-way analysis of variance (ANOVA) followed by Tukey´s multiple comparison was used to test differences between groups; ***p < 0.001; ns not significant (about 100 vacuoles were evaluated per experiment). D, E, F, Kruskal–Wallis test followed by Dunn’s multiple comparisons was used to test differences between groups; ****p < 0.0001; ***p < 0.001; **p < 0.01; *p < 0.05; ns not significant (30 vacuoles were evaluated per experiment). (See S2 and S14 Figs for data related to A, B, C, D, E, F, S5 Fig for data related to A and S6 Fig for data related to C, D, E).

In mice, an essential part of *T*. *gondii* control is mediated by IFNγ-inducible IRG and Guanylate-Binding Proteins (GBP proteins). Accumulation of IRG and GBP proteins at the PVM leads to vacuolar disintegration and parasite and host cell death. To determine if ROP39 contributes to counteract the IFNγ response, parasite clearance was determined in cells stimulated with IFNγ for 24 h or not and subsequently infected with indicated *T*. *gondii* strains for seven days. In plaque assays, no differences between strains were observed in absence of IFNγ indicating no defect in replication of the mutant *T*. *gondii* strains compared with RHΔ*hxgprt* (Figs 5A and S5). The single deletion mutant RHΔ*rop18* but not RHΔ*rop39* showed a significant increase in susceptibility to IFNγ compared with wt *T*. *gondii* infections. This susceptibility was significantly increased in case of the double deletion mutant RHΔ*rop18*/*rop39* (Figs 5 and S5). IRG effectors such as Irga6, Irgb6, Irgb10 and Irgd are prevented from premature activation in uninfected cells by association with IRG regulators Irgm1, Irgm2 or Irgm3 [23–26]. In the absence of Irgm1 and Irgm3, these IRG effectors are mislocalised to lipid droplets [23,25] resulting in uncontrolled parasite replication. We infected *Irgm1*/*Irgm3* ko and wt mice with *T*. *gondii* RHΔ*rop18*/*rop39* to evaluate any contribution of ROP39 to parasite virulence beyond the IRG resistance system. *T*. *gondii* RHΔ*rop18*/*rop39* that is attenuated in wt mice at a dose of 1x10^3^ parasites (Figs 4C and 5B) retrieve its full virulence potential in absence of Irgm1/Irgm3 (Fig 5B). These results indicate that ROP39 primarily counteracts the IFNγ-dependent IRG response.

To determine whether attenuated virulence of RHΔ*rop18*/*rop39* is reflected by the vacuolar IRG loading phenotype, we determined Irga6 frequencies and intensities 2 h post infection with *T*. *gondii* single and double ko strains. Frequencies of Irga6 were similarly increased in single and double ko strain infections compared to RHΔ*hxgprt* (Fig 5C) and therefore do not reflect attenuation of RHΔ*rop18*/*rop39 in vivo* (Figs 4C and 5B) and *in vitro* (Fig 5A). However, Irga6 intensities were not only increased significantly at RHΔ*rop39*-derived vacuoles compared with RHΔ*hxgprt* (Fig 5D) but also at RHΔ*rop18*/*rop39*-derived vacuoles compared with RHΔ*rop18* (Fig 5E). Both Irga6 frequencies and intensities, were restored to levels of the parental strains (Fig 5C–5E) after complementation of single and double ko strains (S6 Fig) with Myc-tagged ROP39 (Fig 4B and 4D).

We then directly compared the impact of ROP39 and ROP18 on Irga6 amounts at the PVM. Intensities of Irga6 were significantly increased in RHΔ*rop18* compared with RHΔ*rop39* infections (Fig 5F). These results might explain the virulence differences of single *T*. *gondii* ko strains RHΔ*rop18* and RHΔ*rop39 in vivo* (Fig 4A). However, at this point the molecular mechanism for ROP39-mediated inhibition of vacuolar Irga6 accumulation still remained elusive.

### ROP39 is an Irgb10-specific parasite effector

IRG proteins load onto the PVM in a defined hierarchy that has been established previously. Members higher in hierarchy (e.g. Irgb10 and Irgb6) influence loading of members lower in hierarchy (e.g. Irga6) [31]. Therefore, we considered another IRG protein as target of ROP39.

**Fig 6:**
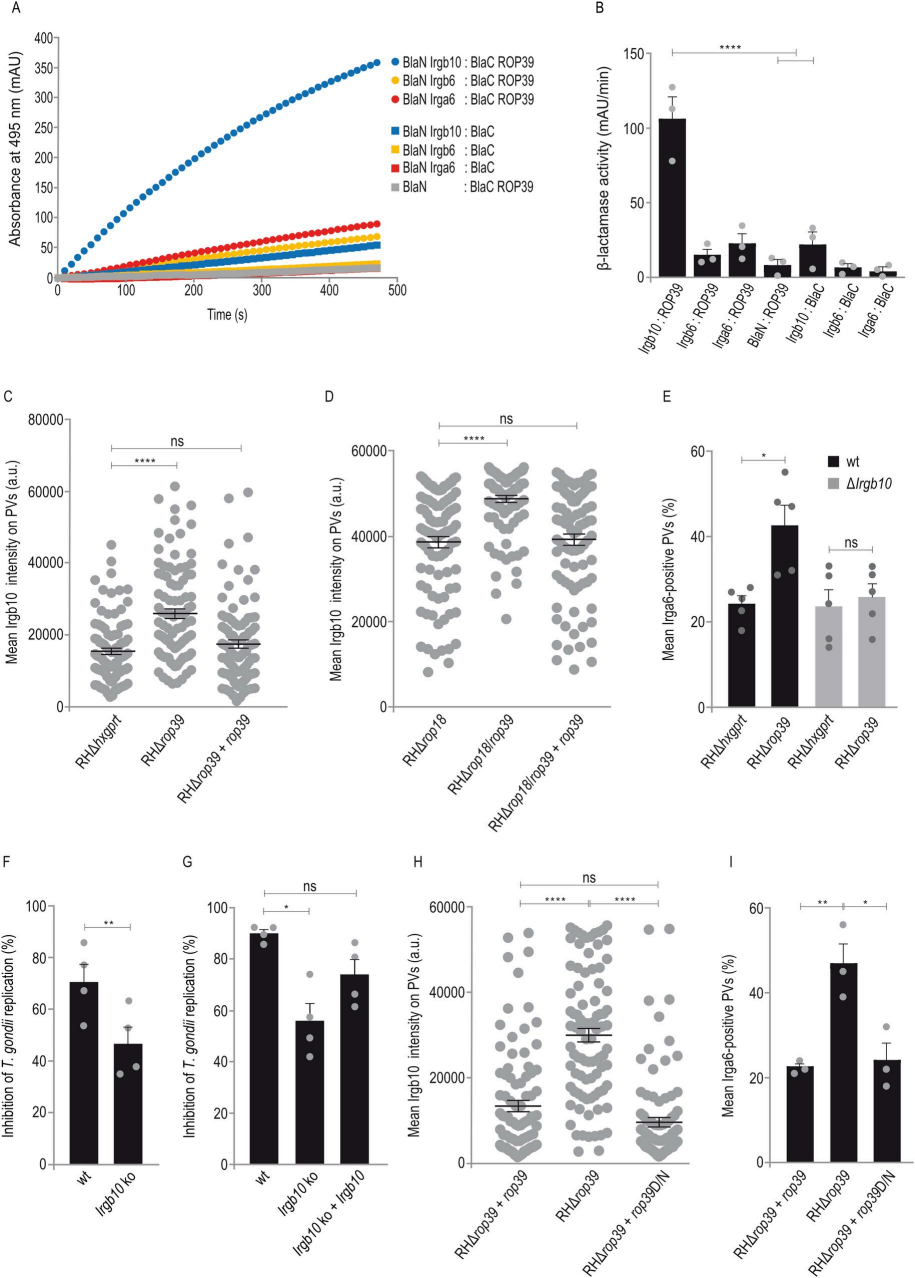
ROP39 is an Irgb10-specific parasite effector. A, B, Protein-fragment complementation assay. Proteins were fused to N-terminal (BlaN) or C-terminal (BlaC) fragments of the reporter protein TEM-1 β-lactamase. The increase in absorbance measured at 495 nm indicates restoration of β-lactamase activity after protein:protein-interaction. A, The kinetic of the β-lactamase reaction is shown for one representative experiment. B, Significant binding of ROP39 to Irgb10 but not Irga6 or Irgb6. Error bars indicate the mean and standard deviation of three independent experiments. One-way analysis of variance (ANOVA) followed by Tukey’s multiple comparison was used to test differences between groups; ****p < 0.0001. C, D, E, H, I, Mouse embryonic fibroblasts were stimulated with IFNγ for 24 h and infected for 2 h before cells were prepared for immunofluorescence. C, D, H, Intensities of Irgb10-positive vacuoles. Error bars indicate the mean and standard error of the mean (SEM) of three independent experiments. Kruskal–Wallis test followed by Dunn’s multiple comparisons was used to test differences between groups; ****p < 0.0001; ns not significant (30 vacuoles were evaluated per experiment). E, I, Frequencies of Irga6-positive vacuoles. Error bars indicate the mean and standard error of the mean (SEM) of three independent experiments. One-way analysis of variance (ANOVA) followed by Tukey´s multiple comparison was used to test differences between groups; **p < 0.01; *p < 0.05; ns not significant (about 100 vacuoles were evaluated per experiment). F, G, Mouse fibroblasts were induced with IFNγ for 24 h and infected with *T*. *gondii* ME49 expressing firefly luciferase (ME49-Luc) for 48 h. Intracellular parasite growth was determined as described in Materials and methods. Error bars indicate the mean and SEM of four independent experiments. Student´s t-test was used for two-group comparisons; **p < 0.01 (F). One-way ANOVA followed by Tukey’s multiple comparison was used to test differences between groups; *p < 0.05; ns not significant (G). (See S2 and S14 Figs for data related to C, D, E, H, I, S6 Fig for data related to C, D, H, I, S8 Fig for data related to C, D, S9 Fig for data related to E, F, G and S11 Fig for data related to C, E, H, I).

Recently, interaction of ROP5B and Irgb2-b1 was demonstrated in the Protein-fragment complementation assay (PCA) [43]. This assay is based on TEM-1 β-lactamase (Bla) of *Escherichia coli*, with two fragments of the reporter protein (Bla) are fused to two putative interaction partners. The individual Bla fragments are non-functional unless their proximity is restored by interaction of the fusion proteins. Using this approach, ROP39 was investigated in terms of binding to IRG effectors Irga6, Irgb6 and Irgb10. Only cotransfection of ROP39 and Irgb10 restored β-lactamase activity (Figs 6A and S7). Three independent biological replicates reveal a significant interaction of ROP39 with Irgb10 (Fig 6B).

Direct binding of ROP39 would suggest an impact on vacuolar Irgb10 accumulation, but no differences in numbers of Irgb10-positive vacuoles were found following infection with RHΔ*rop39* in comparison with RHΔ*hxgprt* (Fig 3A). However, when we determined the amount of Irgb10 at individual Irgb10-positive vacuoles 2 h post infection with the respective *T*. *gondii* strains, the mean Irgb10 signal intensities were significantly increased at RHΔ*rop39*-derived vacuoles compared with RHΔ*hxgprt* (Figs 6C and S8A), as well as at RHΔ*rop18*/*rop39*-derived vacuoles compared with RHΔ*rop18* (Figs 6D and S8B). Irgb10 intensities were restored to levels of the parental strains (RHΔ*hxgprt* in C and RHΔ*rop18* in D) after complementation of single and double ko strains with Myc-tagged ROP39. The loading hierarchy of IRG proteins implies that PVM accumulation of Irga6 depends on preceding vacuolar Irgb10 localisation [31]. To confirm an Irgb10-specific function of ROP39, we determined Irga6-positive vacuoles in *Irgb10* ko [50] in comparison to wt cells. Whereas Irga6 frequencies are significantly increased in wt cells in absence of ROP39, no differences between RHΔ*hxgprt* and RHΔ*rop39* infections could be observed in *Irgb10* ko cells (Fig 6E). Equal expression levels of effector IRG proteins in wt and ko cells were confirmed by Western blot analysis from detergent cell lysates stimulated with IFNγ (S9 Fig). These results confirm ROP39 as an Irgb10-specific parasite effector and demonstrate that ROP39-mediated alteration of Irga6 frequencies depends on Irgb10. Additional evidence that Irga6 loading onto the PVM depends on Irgb10 is provided by identification of Irgb10:Irga6 dimer formation (S10 Fig). IRG protein accumulation at the PVM is required for vacuolar disruption and subsequent parasite death [8]. Direct targeting by ROP39 and inhibition of PVM accumulation therefore suggests that Irgb10 is required for parasite control. When we determined replication of *T*. *gondii* ME49 expressing firefly luciferase (ME49-Luc), inhibition of *T*. *gondii* replication was indeed significantly diminished in *Irgb10* ko relative to wt cells upon stimulation with IFNγ (Fig 6F). This defect of *Irgb10* ko cells was resolved after complementation with Irgb10 (Fig 6G). The inhibition of replication did not reach wt levels probably because among all IRG effector proteins, expression levels of Irgb10 are lower in complemented compared with wt cells (S9 Fig). To investigate whether ROP39-mediated inhibition of vacuolar IRG protein accumulation depends on Irgb10 phosphorylation, we complemented *T*. *gondii* RHΔ*rop39* with *rop*39D/N, a ROP39 kinase dead mutant carrying—in analogy to ROP18 [51]—an exchange of the key catalytic aspartate to asparagine (ROP39D/N) at amino acid position 406 (RHΔ*rop39*+*rop39*D/N, Fig 2B). Using this approach, we determined Irgb10 intensities and Irga6 frequencies at the PVM after stimulation with IFNγ for 24 h. Generation of *T*. *gondii* RHΔ*rop39*+*rop39*D/N was confirmed by Sanger sequencing (S11A Fig), Western blot (S11B Fig) and diagnostic PCR (S6 Fig). Compared to RHΔ*rop39*, Irgb10 intensities (Fig 6H) and Irga6 frequencies (Fig 6I) were similarly reduced upon infection with RHΔ*rop39*+*rop39* and RHΔ*rop39*+*rop39*D/N. In summary, these results indicate that contribution of ROP39 to *T*. *gondii* virulence is not mediated by Irgb10 phosphorylation.

### ROP39 inhibits the formation of Irgb10 dimers

**Fig 7:**
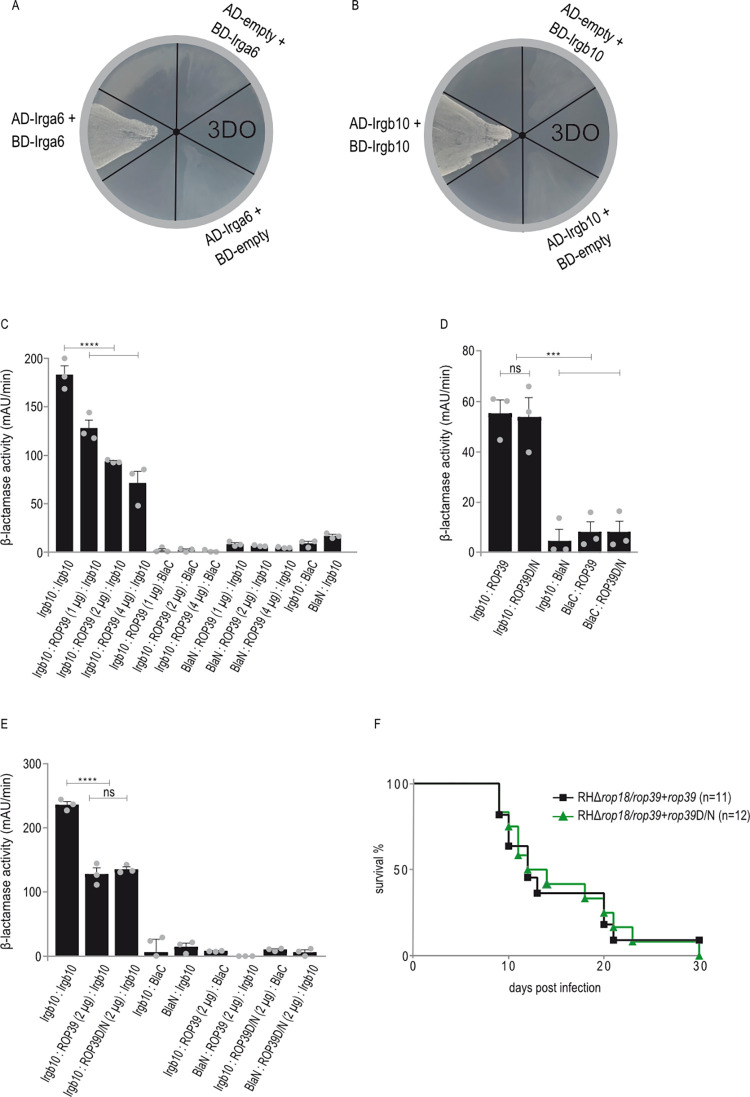
ROP39 inhibits the formation of Irgb10 dimers. A, B, Yeast Two-Hybrid. Colony growth under 3DO conditions demonstrates Irga6 (A) and Irgb10 (B) dimerization. Proteins were expressed either as fusion to a transcriptional activation (AD) or DNA-binding (BD) domain. C, D, E Protein-fragment complementation assay. Proteins were fused to N-terminal (BlaN) or C-terminal (BlaC) fragments of the reporter protein TEM-1 β-lactamase. The increase in absorbance measured at 495 nm indicates restoration of β-lactamase activity after protein:protein-interaction. C, Dimerization of Irgb10 is significantly inhibited in the presence of ROP39. D, ROP39 wt and the kinase dead version (ROP39D/N) bind to Irgb10. E, Dimerization of Irgb10 is significantly inhibited in the presence of wt ROP39 and ROP39D/N. C, D, E, Error bars indicate the mean and standard deviation of three independent experiments. One-way analysis of variance (ANOVA) followed by Tukey’s multiple comparison was used to test differences between groups; ****p < 0.0001; ***p < 0.001; ns not significant. F, Survival of BL/6 wt mice that were intraperitoneally infected with 200 freshly prepared tachyzoites of *T*. *gondii* RHΔ*rop18*/*rop39*+*rop39* or RHΔ*rop18*/*rop39*+*rop39*D/N. (See S2, S5 and S14 Figs for data related to F, S12 Fig for data related to C, D, E and S13 Fig for data related to C).

Irga6 forms GTP-dependent oligomers and hydrolyses GTP in a cooperative manner *in vitro* and *in vivo* [52,53]. At the PVM, Irga6 is in the active, GTP-bound state [52]. These studies suggest that IRG protein oligomerisation is a prerequisite for disruption of the *T*. *gondii*-derived PVM. *T*. *gondii* ROP18 is an Irga6-specific kinase that phosphorylates conserved threonine residues T102/T108, thereby inhibiting Irga6 oligomerisation [39]. Consequently, PVM integrity is preserved allowing uncontrolled parasite replication. IRG protein oligomerisation has been demonstrated for Irga6 [53]. We therefore investigated Irgb10:Irgb10 and—as positive control—Irga6:Irga6 interaction in the YTH assay. Colony growth on selective medium clearly demonstrates formation of Irga6 (Fig 7A) and Irgb10 homodimers (Fig 7B). We then asked whether ROP39 binding to Irgb10 (Fig 6B) affects Irgb10 dimerisation. Dimerisation of Irgb10 fused to different fragments of the reporter protein (Bla) was investigated in absence and presence of ROP39 that was not fused to any Bla fragment. Dimerisation of Irgb10 was significantly inhibited in the presence of ROP39 and the inhibitory effect increased with increasing ROP39 amounts (Figs 7C and S12A). Irgb10 dimerisation was unaffected by the presence of ROP5A or DsRed as inert protein controls (S13 Fig). To investigate whether kinase activity of ROP39 is necessary for inhibition of Irgb10 dimerisation, we first directly compared binding of ROP39 wt and ROP39D/N to Irgb10. Restoration of β-lactamase activity indicated no difference between wt and kinase dead ROP39 (Figs 7D and S12B). We then directly compared the inhibitory effect of ROP39 wt and ROP39D/N on Irgb10 dimerisation. Dimerisation of Irgb10 was inhibited to the same extent in the presence of wt or kinase dead ROP39 (Figs 7E and S12C). To determine whether a putative kinase activity of ROP39 is important for *T*. *gondii* virulence *in vivo*, we complemented the double deletion mutant RHΔ*rop18*/*rop39* with *rop39*D/N (RHΔ*rop18*/*rop39*+*rop39*D/N, S6 Fig) and compared virulence directly with RHΔ*rop18*/*rop39*+*rop39*. No difference between stains was detectable at an inoculum of 200 tachyzoites in BL/6 animals (Fig 7F). Generation of *T*. *gondii* RHΔrop18/*rop39*+*rop39*D/N was confirmed by Sanger sequencing (S14A Fig) and Western blot (S14B Fig).

## Discussion

For successful transmission, *T*. *gondii* has to balance the host immune response to allow establishment of chronic infection. In one of its evolutionarily most important intermediate hosts, *Mus musculus*, the control of parasite virulence depends on a balanced relationship between Immunity-Related GTPases (IRG proteins) [54] providing resistance, and effector proteins that are secreted by the parasite into the host cell cytosol [27]. Among all effectors identified so far, the products of the *rop5* locus were shown to have the largest impact on virulence [6,29] and we have demonstrated recently that ROP5 isoform B (ROP5B) is largely responsible [43]. In search of novel interaction partners for ROP5B, we identified ROP39. ROP39 belongs to the ROP2 family of kinases and pseudokinases [55] and its contribution to recruit host cell mitochondria to the PVM in human cells was demonstrated recently [48]. We could here demonstrate that ROP39 derived from *T*. *gondii* type I specifically binds to Irgb10. Biochemical studies with Irga6 *in vitro* suggest that oligomerisation of IRG proteins at the parasitophorous vacuolar membrane (PVM) of *T*. *gondii* leads to membrane disruption and parasite clearance [8]. We show that ROP39 binding blocks Irgb10 dimerisation and inhibits its accumulation at the *T*. *gondii*-derived PVM. A distinct trait of the IRG system is that individual members load onto the *T*. *gondii* PVM in a defined hierarchy. Vacuolar accumulation of pioneer IRG proteins Irgb10 and Irgb6 is followed by loading of IRG members lower in hierarchy [31]. This might explain why ROP39-mediated reduction of the overall amount of Irgb10 at the PVM compromises vacuolar Irga6 but not Irgb6 accumulation. How an increase in Irgb10 intensities but not frequencies in absence of ROP39 should lead to an increase in Irga6 frequencies (Fig 3A) is not immediately clear. It is plausible that the overall amount of Irgb10 is actually increased at all vacuoles and that these elevated Irgb10 levels allow recruitment and visualization of Irga6 but are still below the limit of detection for the anti Irgb10 antibody. However, in *Irgb10* ko cells, RHΔ*rop39* infections do not entail increased Irga6 frequencies relative to RHΔ*hxgprt* anymore (Fig 6E). These data demonstrate that ROP39 is Irgb10-specific and alteration of vacuolar Irga6 accumulation depends on Irgb10 and in that way illustrate the complexity of the IRG protein network necessary for *T*. *gondii* control. The importance of ROP39-mediated inhibition of Irgb10 for parasite virulence is demonstrated by significantly diminished *T*. *gondii* ME49 control in the absence of Irgb10 (Fig 6F and 6G). There is a discrepancy between our results and results obtained in a previous study [17]. In this context it should be noted that *T*. *gondii* ME49 seems to be more virulent than Prugnaud (Pru) [56] what might explain efficient control of Pru even in the absence of *Irgb10* in [17]. Other explanations could be different times of infection, amounts of IFNγ and/or MOIs. However, after complementation of *Irgb10* ko cells with Irgb10, replication is restored to wt levels confirming Irgb10-related *T*. *gondii* ME49 control in our experiments (Fig 6G).

Whereas single deletion of *rop39* in a type I genetic background had almost no effect on *T*. *gondii* virulence *in vivo*, the double deletion mutant RHΔ*rop18*/*rop39* was significantly attenuated compared with RHΔ*rop18*. The combined activities of ROP18 and ROP39 were reflected by increased vacuolar Irgb10 and Irga6 recruitment to the PVM and IFNγ-dependent parasite clearance *in vitro*. These findings are consistent with a synergistic function of both virulence effectors that is revealed by severe attenuation of virulence in case of the double deletion mutant compared with individual mutations. The avirulent *rop18*/*rop39* ko phenotype was reverted in *Irgm1*/*Irgm3* ko mice. In these mice, the IRG system is virtually lost [24]. Therefore, although it cannot be completely excluded that ROP39 has additional targets, the predominant function of this effector is disturbance of the IRG system by directly targeting Irgb10.

Two highly conserved threonine residues, T102 and T108, within the switch 1 region of the G-domain have been identified as sensitive targets for IRG protein inhibition by ROP18- and ROP17-mediated phosphorylation [34,35,39]. In addition to direct interactions, protein kinases and their substrates interact through adaptors or scaffolds that serve as platforms to assemble all necessary components [57]. Defining the composition of such scaffolds is relevant to understand the regulatory mechanisms of IRG-related *T*. *gondii* effectors. We have shown that the Irgb10-specific ROP39/ROP5/GRA7 complex and its molecular interactions mirror the composition of the Irga6-specific ROP18/ROP5/GRA7 [37] and Irgb6-specific ROP17/ROP5/GRA7 [34] kinase complexes. In a plausible scenario, ROP5 is the central element that is scaffolding other effectors to accomplish IRG-specific complex formation at the PVM upon *T*. *gondii* infection. In that way, the physiological local concentrations of the reactants are increased to accomplish efficient and specific kinase activity. In the present study, we were unsuccessful in identification of Irgb10-specific phosphopeptides by mass spectrometry after immunoprecipitation from *T*. *gondii* type I-infected cells (S15 Fig). These results are consistent with results from our previous study, where *T*. *gondii* type I-mediated phosphorylation of endogenous Irgb10 was hardly detectable [39]. Moreover, the kinase dead mutant carrying an exchange of one key catalytic aspartic acid (D406) to asparagine (N406) within the ROP39 sequence (ROP39D/N) bound Irgb10 and inhibited its dimerisation like the wt ROP39. After complementation of *T*. *gondii* RHΔ*rop39* with *rop39*D/N, the Irgb10 and Irga6 loading phenotypes were essentially the same as in *T*. *gondii* wt strain infections. The latter result demonstrates that kinase activity of ROP39 is dispensable for inhibition of the IRG resistance system. To test the contribution of a putative ROP39 kinase activity to parasite virulence *in vivo*, we complemented the double deletion mutant RHΔ*rop18*/*rop39* with *rop39*D/N (RHΔ*rop18*/*rop39*+*rop39*D/N). No virulence differences compared with RHΔ*rop18*/*rop39*+*rop39* were detectable. It remains to be determined whether ROP39 binding traps Irgb10 in the inactive GDP-bound conformation and thereby blocks GTP-dependent oligomerisation or whether—not mutually exclusive—it physically masks an interface necessary for dimerization and in that way resembles the ROP5-mediated inhibition of Irga6 oligomerisation [36]. However, the catalytic triad of amino acid residues that are considered to be required for catalytic activity is preserved within the ROP39 sequence [45,46] and our results do not exclude kinase activity of ROP39 *per se*. Future experiments will clarify whether ROP39 is indeed an active kinase and whether enzymatic activity might be important for *T*. *gondii* virulence upon infection of different intermediate hosts than *Mus musculus*.

The *rop18*/*rop39* double deletion mutant is not as impaired as the *rop5* ko strain and in that way resembles the *T*. *gondii* RHΔ*rop17*/*rop18* strain [34]. Whether virulence of the triple deletion mutant *rop17*/*rop18*/*rop39* would phenocopy the *rop5* ko phenotype or whether other *T*. *gondii* ROP5-dependent effectors targeting additional IRG protein family members exist cannot be answered conclusively yet. The synergistic effect of *T*. *gondii* effectors (ROP18/ROP39, this study; [32,34]) complicates the evaluation of single deletion mutants. We demonstrate that identification of binding partners of ROP5B provides an alternative approach to discover novel effectors important for *T*. *gondii* virulence. In analogy to IRG proteins, these effectors might be specific for inactivation of the Guanylate Binding Proteins (GBP). GBP proteins are essential for normal parasite control [58] but detailed functional analysis of *T*. *gondii* virulence effectors in context of GBP protein inactivation is not yet available. Apparently, both families of GTPases cannot be considered as single entities but accumulation of IRG and GBP proteins at the PVM is rather interdependent [17,59–62]. Although no data demonstrating the molecular details of such an interplay of both families of GTPases are available, this would explain reduction of GBP protein accumulation in context *T*. *gondii* ROP18 [59,63–65]. However, it is conceivable that *T*. *gondii* has evolved GBP-specific effector proteins and these effectors might as well indirectly affect the accumulation of certain IRG proteins. The identification of these effectors, their dependency on ROP5—in case they exist—and the mechanism underlying a possible interplay of IRG and GBP proteins remains an important area for future research.

The multilayered survival strategy of *T*. *gondii* is embodied in a family of polymorphic kinases and pseudokinases. These effectors assemble in multiprotein complexes and cooperate to combat members of the highly divergent family of IRG proteins at the PVM. Since virulence leads to early death of the host and consequent interruption of parasite transmission it is reasonable to assume that the biological targets of *T*. *gondii* effectors are host resistance molecules that are so effective that they can induce sterile immunity. For the time being, no IRG proteins capable of this level of effect are known in the mouse but may be present in other evolutionarily significant hosts. Our study identifies ROP39 as an essential element of a novel *T*. *gondii* complex important for inactivation of Irgb10. The complex dynamics between these two systems of parasite virulence and host cell resistance documents the coevolutionary relationship of *T*. *gondii* and *Mus musculus*, an important intermediate host for transmission.

## Materials and methods

### Ethics statement

All animal experiments were performed in compliance with the German animal protection law (TierSchG). Mice were handled in accordance with good animal practice as defined by FELASA and the national animal welfare body GV-SOLAS. The animal welfare committees of the universitiy of Freiburg as well as the local authorities (Regierungspräsidium Freiburg; Landesamt für Natur, Umwelt und Verbraucherschutz Nordrhein—Westfalen; Behörde für Soziales, Familie, Gesundheit und Verbraucherschutz, Hamburg) approved all animal experiments.

### Cell culture

Human embryonic kidney (HEK) 293 SV40 T-antigen (T) cells (HEK293T, ATTC; CRL-3216), mouse embryonic fibroblasts (MEF) derived from C57BL/6 mice and human foreskin fibroblasts (HS27, ATCC; CRL-1634) were maintained by serial passage in DMEM, high glucose (Invitrogen Life Technologies) supplemented with 2 mM L-glutamine, 100 U/ml penicillin, 100 mg/ml streptomycin (PAA) and 10% fetal calf serum (FCS, anprotec). All cells were mycoplasma-free and regularly tested by PCR [66].

### Animal strains and infection conditions

C57BL/6 wt and *Irgm1*/*Irgm3* ko mice lacking the regulatory IRG proteins Irgm1 and Irgm3 were obtained from certified breeders or in-house colonies. Female and male mice with ages ranging from 6 to 8 weeks were infected intraperitoneally (i.p.) with 200 μl of sterile PBS containing freshly harvested tachyzoites of indicated *T*. *gondii* strains. Survivors were sacrificed at the indicated days post-infection and tested for sero-conversion using the Toxocell latex kit and by ELISA [67].

### Propagation of *T*. *gondii*

Tachyzoites of *T*. *gondii* RHΔ*hxgprt*, RHΔ*rop5*, RHΔ*rop18*, RHΔ*rop39*, RHΔ*rop39*+*rop39*, RHΔ*rop39*+*rop39*D/N, RHΔ*rop18/rop39*, RHΔ*rop18/rop39*+*rop39*, RHΔ*rop18/rop39*+*rop39*D/N and ME49-Luc were cultivated in confluent monolayers of human foreskin fibroblasts (HS27), harvested and immediately used for *in vitro* or *in vivo* infections or lysed for subsequent pull-down experiments or Western blot analysis.

### Postnuclear lysate preparation from free tachyzoites and infected cells

100 x 10^6^ free *T*. *gondii* tachyzoites or 2.5 x 10^5^ MEFs seeded in 6 well-plates, stimulated with 200 U/ml IFNγ for 24 h, subsequently infected for 2 h with *T*. *gondii* at a MOI of 10 and washed 2x with PBS were lysed in NP-40-lysis buffer (0.1% NP-40, 150 mM NaCl, 20 mM Tris/HCl (pH 7.6), 5 mM MgCl_2_ supplemented with protease inhibitors) for 2 h under constant rotation at 4°C. Postnuclear lysates were subjected to pull-down or Western blot analysis.

### Pull-down analysis

Purified GST or GST-ROP39 was mixed with 100 μl 1:1 bead suspension of Glutathione Sepharose 4B resin in 600 μl PBS containing 2 mM DTT for 1 h under continuous rotation at 4°C. Beads were washed 3x with ice-cold lysis buffer without detergent and incubated with freshly prepared postnuclear lysates of *T*. *gondii* tachyzoites (20 x 10^6^) o/n in 150 mM NaCl, 20 mM Tris/HCl (pH 7.6), 5 mM MgCl_2_, 2 mM DTT supplemented with protease inhibitors at 4°C. Beads were washed 3x with ice-cold NP-40-lysis buffer and either stored at -80°C or immediately boiled in sample buffer (80 mM Tris/HCl (pH 6,8), 5 mM EDTA, 4% SDS, 34% sucrose, 40 mM DTT, 0.002% bromphenol blue) for 5 min at 95°C and subjected to SDS-PAGE and Western Blot.

### Preparation of *T*. *gondii* DNA

Genomic DNA (gDNA) was prepared from *T*. *gondii* RHΔ*hxgprt*, RHΔ*rop18*, RHΔ*rop39*, RHΔ*rop39*+*rop39*, RHΔ*rop39*+*rop39*D/N, RHΔ*rop18/rop39*, RHΔ*rop18/rop39*+*rop39* and RHΔ*rop18*/*rop39*+*rop39*D/N tachyzoites using the Quick DNA miniprep kit.

### Generation of *T*. *gondii* knockout and complemented strains

For generation of RHΔ*rop39* and RHΔ*rop18/rop39*, 10^7^ freshly egressed RHΔ*hxgprt* or RHΔ*rop18* parasites were pelleted for 15 min at 1,000 g, washed and resuspended in cytomix buffer (120 mM KCl, 150 μM K_2_HPO_4_/KH_2_PO_4_, 10 mM Hepes, 2 mM EGTA, 5 mM MgCl_2_, 3 mM ATP, 3 mM glutathione). Electroporation was performed in a 4 mm cuvette with 20 μg of the gRNA-specific CRISPR/Cas9 vector (pU6-*rop39*) and 7 μg of the respective DHFR-TS cassette in a final volume of 800 μl (2 pulses of 1.7 kV for 0.18 ms at 5 sec intervals). After growth for 24 h in HFF monolayers, pyrimethamine (3 μM) was used to select resistant transformants. Serial dilutions were performed in the presence of pyrimethamine to select the clonal RHΔ*rop39* and RHΔ*rop18/rop39* lines.

RHΔ*rop39*+*rop39*, RHΔ*rop39*+*rop39*D/N, RHΔ*rop18/rop39*+*rop39* and RHΔrop18/*rop39*+*rop39*D/N were generated by electroporation of RHΔ*rop39* or RHΔ*rop18/rop39* parasites with 20 μg of the CRISPR/Cas9 plasmid pU6-UPRT and 10 μg of linearized pUPRT-RON5-*rop39*-Myc or pUPRT-RON5-*rop39*D/N-Myc and subsequent selection with 5 μM FUDR (5-Fluoro-2′-deoxyuridine) in HFF cells. Serial dilutions were performed in the presence of 5 μM FUDR.

### Plaque assays

C57BL/6 MEFs confluently grown in 6 well-plates in the absence or presence of 100 U/ml IFNγ were infected with 200 freshly harvested tachyzoites of *T*. *gondii* wt and ko strains. 7 days post infection, monolayers were thoroughly washed with PBS and plaque numbers determined after staining with 0,1% crystal violet [67].

### Lentiviral transduction

Gag-pol and env-expressing plasmids were co-transfected with a plasmid encoding *Irgb10* in HEK293T cells that have been grown to a density of 70% in a 10 cm plate. The medium was exchanged 24 h post transfection and cells grown for additional 24 h before the supernatant was filtered and transferred to *Irgb10* ko cells that have been seeded 1 day before in a 6 cm plate. After 24 h, transduced cells were selected with 1–5 μg puromycin in appropriate cell culture flasks for several days.

### Expression and purification of recombinant proteins

GST or GST-ROP39 were expressed from pGEX-4T-2 constructs in *Escherichia coli* BL21 o/n at 18°C in presence of 0,1 mM IPTG. Cells were lysed in PBS/2 mM DTT/0,1% Triton X-100 using a French Press. Lysates were cleared by centrifugation at 50,000 g for 60 min at 4°C and loaded on a GSTrap FF Glutathione Sepharose affinity column in PBS/2 mM DTT/0,1% Triton X-100.

### Immunocytochemistry

Fixation and staining of MEFs grown on coverslips was performed as described earlier [39]. Microscopy and image analysis was performed blind on coded slides essentially according to [31]. Intracellular parasites were identified from the pattern of *T*. *gondii* GRA7 staining.

### Yeast two-hybrid assay

Five single colonies of *Saccharomyces cerevisiae* strain PJ69-4α grown on Yeast Peptone Dextrose Agar (YPDA) plates were resuspended in 100 μl transformation buffer (50% PEG 3350, 0.2 M LiAc, 0.5 mg/ml ss DNA, 0.1 M DTT) and incubated with 1 μg of plasmid DNA (pGAD-C3, pGBD-C3, pGAD-T7 or pGBD-T7 containing the indicated genes) for 30 min at 42°C. Cotransformants were selected by plating on double dropout media (SD/-Leu/-Trp). Colonies grown on double dropout media were replica plated on double dropout media before OD_600_ measurement of single colonies resuspended in liquid triple dropout media (SD/-Leu/-Trp/-His). Same amount of material was plated on triple dropout media containing 0.5 mM 3-AT and incubated for 5 to 14 days at 30°C.

### Protein-fragment complementation assay

The Protein-fragment complementation Assay (PCA) is based on split TEM-1 β-lactamase (encoded by the *Bla* gene) of *Escherichia coli* [68]. Two fragments of the reporter protein were fused to two putative interaction partners. The individual β-lactamase fragments are non-functional unless proximity upon interaction of the fusion proteins is restored. 7.5 x 10^5^ HEK293T cells seeded in 6 well-plates were co-transfected with 1 to 6 μg respective plasmid DNA using Lipofectamine 3000 following the manufacturer´s instructions. 24 h post transfection, cells were trypsinized, washed 1x with PBS and resuspended in 100 μl passive lysis buffer (1x) containing protease inhibitor cocktail. After 60 min incubation on ice and centrifugation for 30 min at 15,000 g and 4°C, 50 μl of supernatants were mixed with 15 μl nitrocefin and 135 μl PBS in a 96 well-plate. The β-lactamase-mediated hydrolysis of nitrocefin was measured by the change of absorbance at 495 nm at intervals of 8–9 seconds for 50 cycles.

In the presence of a standard substrate concentration, the actual nitrocefin hydrolysis rate is dependent on the amounts of reconstituted β-lactamase, consequently on the interaction between the fusion proteins. Therefore, to determine the strength of the interaction, the nitrocefin hydrolysis rates, expressed in mAU/min, were calculated for the linear phase of the reaction and compared to each other and the background rates, which were observed upon transfection of the respective fusion proteins alone [68,69]. All PCA assays were carried out three times and the differences between average hydrolysis rates were compared to evaluate the strength of the interactions.

### *T*. *gondii* replication assay

Wt or *Irgb10* ko tail fibroblasts were grown for 24 h in the presence of 200 U/ml IFNγ or left untreated and subsequently infected with *T*. *gondii* ME49 expressing firefly luciferase (ME49-Luc) at MOI 0,25. At 48 h post infection, cells were lysed and Luc activity was determined by bioluminescence. Percental inhibition of *T*. *gondii* replication was defined as follows: 100 - (mean IFNγ-stimulated/mean unstimulated)*100.

### Bioinformatics and sequence analysis

The secondary structure of *T*. *gondii* type I ROP39 was predicted by PSIpred (UCL department of computer science, http://bioinf.cs.ucl.ac.uk/psipred/). The 3D structure of ROP39 was modeled from the motif SLLD using the trRosetta server (https://github.com/gjoni/trRosetta). The amphipathic character of the N-terminal α-helix of ROP39 was predicted with HeliQuest (https://heliquest.ipmc.cnrs.fr/).

ROP39 sequences of *T*. *gondii* strains ME49 (TGME49_262050), GT-1 (TGGT1_262050) and VEG (TGVEG_262050) were retrieved from the ToxoDB online database (https://toxodb.org/toxo/app). MEGA (Molecular Evolutionary Genetics Analysis) was used to compute a phylogenetic tree by the Maximum Likelihood method.

### Mass spectrometric identification of phosphorylated peptides

Irgb10 was immunoprecipitated from IFNγ-stimulated and *T*. *gondii* RHΔ*hxgprt* infected cells with a rabbit anti Irgb10 antiserum (940/6). Bound proteins were resuspended in lysis buffer (5% SDS, 50mM triethyl ammonium bicarbonate (TEAB), pH 7.5) and eluted by incubation at 95°C, 10 min, and 500rpm. Afterwards samples were sonicated using a Bioruptor device (Diagenode, Liège, Belgium). Samples were centrifuged at 13000g for 8 min and the supernatant used in the following steps. Proteins were reduced using 5 mM tris (2-carboxyethyl) phophine hydrochloride (TCEP) (Sigma) for 10 min at 95°C and alkylated using 10 mM 2-iodoacetamide for 20 min at room temperature in the dark. Following steps were performed using S-Trap micro filters (Protifi, Huntington, NY) following the manufacturer’s procedure. Briefly, a final concentration of 1.2% phosphoric acid and then six volumes of binding buffer (90% methanol; 100 mM triethylammonium bicarbonate, TEAB; pH 7.1) were added to eluted proteins. After gentle mixing, the protein solution was loaded to an S-Trap filter and spun at 2000 rpm for 0.5–1 min. The filter was washed three times using 150 μL of binding buffer. Sequencing-grade trypsin (Promega, 1:25 enzyme:protein ratio) diluted in 20μl digestion buffer (50 mM TEAB) were added into the filter and digested at 47°C for 1 h. To elute peptides, three step-wise buffers were applied: a) 40 μL 50 mM TEAB, b) 40μl 0.2% formic acid in H2O, and c) 50% acetonitrile and 0.2% formic acid in H2O. The peptide solution were combined and dried in a SpeedVac.

For LC-MS/MS measurements one ug of peptides were analyzed on a Q-Exactive Plus mass spectrometer (Thermo Scientific, San Jose, CA) coupled to an EASY-nLC 1000 UHPLC system (Thermo Scientific). The column setup consisted of an Acclaim PepMap 100 C18 column (Thermo Fisher Scientific, Cat. No. 164946) and a 200 cm μPac GEN1 analytical column (PharmaFluidics, 55250315018210) coupled to a Nanospray Flex ion source (Thermo Scientific, ES071) and a fused silica emitter (MS Wil, TIP1002005-5). For peptide separation, a linear gradient of increasing buffer B (0.1% formic acid in 80% acetonitrile, Fluka) was applied, ranging from 5 to 50% buffer B over the first 80 min and from 50 to 100% buffer B in the subsequent 40 min (120 min separating gradient length). Peptides were analyzed in data dependent acquisition mode (DDA). Survey scans were performed at 70,000 resolution, an AGC target of 3e6 and a maximum injection time of 50 ms followed by targeting the top 10 precursor ions for fragmentation scans at 17,500 resolution with 1.6 m/z isolation windows, an NCE of 30 and a dynamic exclusion time of 35 s. For all MS2 scans the intensity threshold was set to 1e5, the AGC to 1e4 and the maximum injection time to 80 ms.

Raw data were analyzed with MaxQuant (v 1.6.17.0) with the built-in Andromeda peptide search engine [70]. The false discovery rate (FDR) at both the protein and peptide level was set to 1%. Two missed cleavage sites were allowed, Phospho (STY) was set as variable modification, and carbamidomethylation of cysteines was set as fixed modification. The match between runs option was selected. For label free quantification the MaxLFQ algorithm was applied using the standard settings. In addition the iBAQ (Intensity Based Absolute Quantification) algorithm was used. Only unique peptides were used for quantification. The Mouse-EBI-reference database was downloaded from https://www.ebi.ac.uk/ on Feb 24th 2020.

### Statistics

All statistical analyses were performed using GraphPad Prism 8.3 software. *P*-values were determined by an appropriate statistical test. Statistical differences in IRG protein intensities between groups at single *T*. *gondii*-derived intracellular vacuoles were determined using Kruskal–Wallis test followed by Dunn’s multiple comparisons. For IRG protein frequencies, One-Way ANOVA followed by Tukey’s multiple comparison or Two-Way ANOVA followed by Sidak’s multiple comparison was used for data with single grouping or two grouping variables respectively. In case of PCA and *T*. *gondii* replication assays, One-way ANOVA followed by Tukey´s multiple comparison was used to test differences between groups or Student´s t-test for two-group comparisons. For *in vivo* experiments, a log-rank Mantel-Cox test was used to test differences between groups. All error bars indicate the mean and standard error of the mean (SEM) of at least three independent experiments. *P*-values; **** p < 0.0001, *** p < 0.001, ** p < 0.01, * p < 0.05, ns no significant.

## Supporting information

S1 Fig (related to Fig 2). ** Multiple amino acid sequence alignment.** Multiple amino acid alignment of ROP39 from indicated *T*. *gondii* strains. Letters in white, non conserved amino acids; letters in blue, conserved amino acids.
(TIFF)

S2 Fig (related to Figs 2, 3, 4, 5, 6 and 7). **Generation of *T*. *gondii* RHΔ*rop39* and RHΔ*rop18*/*rop39* deletion mutants.** A, Schematic representation of specific gRNA-mediated targeting of CRISPR/Cas9 to the endogenous *rop39* locus and integration of the *DHFR* selection cassette carrying *rop39* 5`and 3`UTR homology regions. B, Diagnostic PCR of gDNA prepared from clonal lines demonstrates excision and replacement of the *rop39* exon with the *DHFR* selection cassette.
(TIFF)

S3 Fig (related to Fig 2). **Multiple amino acid sequence alignment.** Multiple amino acid alignment of the N-terminus of indicated *rop* genes from *T*. *gondii* GT-1. Letters in white, non conserved amino acids; letters in blue, conserved amino acids. Red boxes show amphipathic α-helices that mediate membrane targeting in case of ROP2, ROP5, ROP17 and ROP18. In case of ROP39, these sequences are highly degenerate but a single large amphipathic α-helix can be identified instead (yellow).
(TIFF)

S4 Fig (related to Fig 3). **(T102)Irga6 and (T108)Irga6 are no targets of ROP39-mediated phosphorylation.** Phosphorylation of Irga6 at threonine residues T102 ((T102)Irga6) or T108 ((T108)Irga6) is demonstrated upon infection with RHΔ*hxgprt* or RHΔ*rop39* by Western blot using anti p(T102)Irga6- or p(T108)Irga6-specific antibodies.
(TIFF)

S5 Fig (related to Fig 5). **Plaque assay.** Mouse embryonic fibroblasts grown in 6 well-plates in presence or absence of IFNγ (100 U/ml) for 24 h were infected with 200 freshly prepared *T*. *gondii* tachyzoites. A, At 7 d post infection, cell monolayers were stained with crystal violet. One representative experiment is depicted. B, The percentage of plaque number reduction for each *T*. *gondii* strain in presence of IFNγ (+IFNγ) in comparison to non-stimulated conditions (-IFNγ, set at 100%) is shown. Error bars indicate the mean and standard error of the mean (SEM) of four independent experiments. One-way analysis of variance (ANOVA) followed by Tukey´s multiple comparison was used to test differences between groups; ****p < 0.0001, ***p < 0.001; **p < 0.01.
(TIFF)

S6 Fig (related to Figs 4, 5, 6 and 7). **Generation of *T*. *gondii* RHΔ*rop39*+*rop39* and RHΔ*rop18*/*rop39+rop39*.** Schematic representation of CRISPR/Cas9 gRNA-mediated integration of C-terminally Myc-tagged *rop39* including the *ron5* promoter at the endogenous *UPRT* locus.
(TIFF)

S7 Fig (related to Fig 6). **Exemplary schematic representation of a PCA reaction.** Irgb10 and *T*. *gondii* type I ROP39 were fused to N-terminal (BlaN) or C-terminal (BlaC) fragments of the reporter protein TEM-1 β-lactamase respectively. A, Control reaction containing Irgb10 fused to the N-terminal fragment of β-lactamase (BlaN-Irgb10) and an empty plasmid containing only the C-terminal β-lactamase fragment. No restoration of β-lactamase activity is expected. B, Control reaction containing ROP39 fused to the C-terminal fragment of β-lactamase (BlaC-ROP39) and an empty plasmid containing only the N-terminal β-lactamase fragment. No restoration of β-lactamase activity is expected. C, Reaction containing Irgb10 fused to the N-terminal fragment of β-lactamase (BlaN-Irgb10) and ROP39 fused to the C-terminal fragment of β-lactamase (BlaC-ROP39). Upon Irgb10:ROP39-interaction, β-lactamase activity is restored.
(TIFF)

S8 Fig (related to Fig 6). **Representative fluorescent images of Irgb10-positive vacuoles.** A, B, Mouse embryonic fibroblasts have been stimulated with 200 U/ml IFNγ for 24 h and infected with indicated *T*. *gondii* strains at MOI 5. After 2 h, cells were prepared for immunofluorescence analysis as described in Materials and methods. Irgb10 in red (right hand panels), GRA7 in green (middle panels) and overlay (left hand panels) are shown. Pictures for Irgb10-positive vacuoles were taken at the same exposure time. Scale bars, 10 μm.
(TIFF)

S9 Fig (related to Fig 6). **IRG protein expression levels are unaffected in *Irgb10* ko cells.** Western blot of detergent lysates from BL/6 wild-type (wt), *Irgb10* knockout (ko) and Irgb10 complemented ko cells stimulated for 24 h with 200 U/ml IFNγ. The signal representing Irgb10 in wt and complemented cells is lost in *Irgb10* ko cells. Expression levels of all other Immunity-Related GTPases (IRG proteins) are unchanged in ko cells compared with wt and complemented cells. Calnexin serves as loading control.
(TIFF)

S10 Fig (related to Fig 6). **Irgb10 and Irga6 form heterodimers.** Protein-fragment complementation assay. Proteins were fused to N-terminal (BlaN) or C-terminal (BlaC) fragments of the reporter protein TEM-1 β-lactamase (Bla). The increase in absorbance measured at 495 nm indicates restoration of β-lactamase activity after protein:protein-interaction. A, The kinetic of the β-lactamase reaction is shown for one representative experiment. B, Heterodimerisation of Irgb10 and Irga6. Error bars indicate the mean and standard deviation of three independent experiments. One-way analysis of variance (ANOVA) followed by Tukey’s multiple comparison was used to test differences between groups; *p < 0.05.
(TIFF)

S11 Fig (related to Fig 6). **Confirmation of *T*. *gondii* RHΔ*rop39*+*rop39*D/N.** A, Complementation of *T*. *gondii* RHΔ*rop39* with a kinase dead mutant of ROP39 (ROP39D/N) was confirmed by Sanger sequencing of a *rop39*-specific PCR product. The nucleotide exchange from G to A (green) at nt position 1216 leading to an amino acid exchange of the key catalytic aspartate (Asp, D) at position 406 to asparagine (Asn, N) (blue box, upper panel) and the electropherogram (lower panel) is depicted. B, Complementation of RHΔ*rop39* with the kinase dead version of ROP39 (RHΔ*rop39*+*rop39*D/N) is demonstrated in comparison to RHΔ*hxgprt*, RHΔ*rop39* and RHΔ*rop39*+*rop39* by Western blot using a ROP39-specific peptide antiserum (upper panel) or anti-Myc antibody (lower panel).
(TIFF)

S12 Fig (related to Fig 7). **ROP39 inhibits homodimerisation of Irgb10.** Protein-fragment complementation assay. Proteins were fused to N-terminal (BlaN) or C-terminal (BlaC) fragments of the reporter protein TEM-1 β-lactamase (Bla). The increase in absorbance measured at 495 nm indicates restoration of β-lactamase activity after protein:protein-interaction. A, Homodimerisation of Irgb10 is inhibited in the presence of ROP39. B, ROP39 wt and the kinase dead version of ROP39 (ROP39D/N) bind to Irgb10. C, Homodimerisation of Irgb10 is inhibited in the presence of ROP39 wt and the kinase dead version of ROP39 (ROP39D/N). A, B, C The kinetic of the β-lactamase reaction is shown for one representative experiment.
(TIFF)

S13 Fig (related to Fig 7). **PCA inert protein controls.** Protein-fragment complementation assay. Proteins were fused to N-terminal (BlaN) or C-terminal (BlaC) fragments of the reporter protein TEM-1 β-lactamase (Bla). The increase in absorbance measured at 495 nm indicates restoration of β-lactamase activity after protein:protein-interaction. A, Homodimerisation of Irgb10 is not inhibited in the presence of ROP5A as an inert protein control. B, The kinetic of the β-lactamase reaction is shown for one representative experiment. C, Homodimerisation of Irgb10 is not inhibited in the presence of DsRed as an inert protein control. D, The kinetic of the β-lactamase reaction is shown for one representative experiment.
(TIFF)

S14 Fig (related to Fig 7). **Confirmation of *T*. *gondii* RHΔ*rop18*/*rop39*+*rop39*D/N.** A, Complementation of *T*. *gondii* RHΔ*rop18*/*rop39* with a kinase dead mutant of ROP39 (ROP39D/N) was confirmed by Sanger sequencing of a *rop39*-specific PCR product. The nucleotide exchange from G to A (green) at nt position 1216 leading to an amino acid exchange of the key catalytic aspartate (Asp, D) at position 406 to asparagine (Asn, N) (blue box, upper panel) and the electropherogram (lower panel) is depicted. B, Complementation of RHΔ*rop18*/*rop39* with the kinase dead version of ROP39 (RHΔ*rop18*/rop39+*rop39*D/N) is demonstrated in comparison to RHΔ*hxgprt*, RHΔ*rop39*, RHΔ*rop18*/*rop39* and RHΔ*rop18*/*rop39*+*rop39* by Western blot using a ROP39-specific peptide antiserum.
(TIFF)

S15 Fig Mass spectrometry analysis of Irgb10-specific tryptic peptides. Tryptic peptides of immunoprecipitated Irgb10 from IFNγ-induced and RHΔ*hxgprt*-inefected MEFs using an Irgb10-specific antiserum. Identified peptides corresponding to Irgb10 are marked in red (sequence coverage 65,2%). No phosphosite could be revealed in this MS analysis.
(TIFF)

S1 Table Reagents and resources. (PDF)

S2 Table *T*. *gondii* strains used in this study. (PDF)

S3 Table Plasmids and constructs used in this study. (PDF)

S4 Table Oligonucleotides and primers used in this study. (PDF)

S5 Table Immunoreagents used in this study. (PDF)
