# Supplementary figures and images for: ROP39 is an Irgb10-specific parasite effector that modulates acute Toxoplasma gondii virulence
Source: PLoS Pathog. 2023 Jan 5;19(1):e1011003. doi: 10.1371/journal.ppat.1011003 (PMC9848475; doi:10.1371/journal.ppat.1011003)

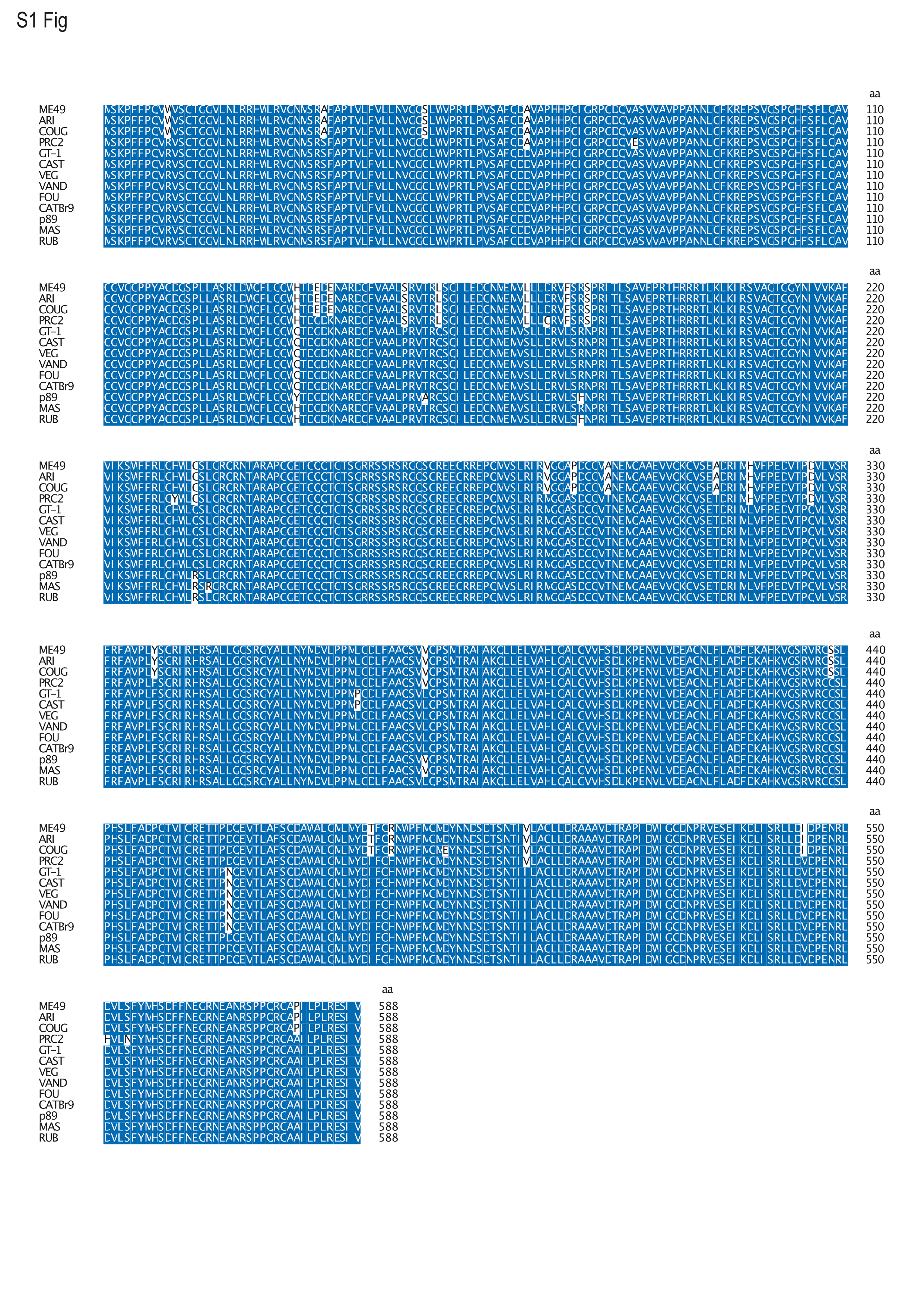

Supplement: S1 Fig — Multiple amino acid sequence alignment. Multiple amino acid alignment of ROP39 from indicated T. gondii strains. Letters in white, non conserved amino acids; letters in blue, conserved amino acids. (TIFF) [file ppat.1011003.s001.tiff]

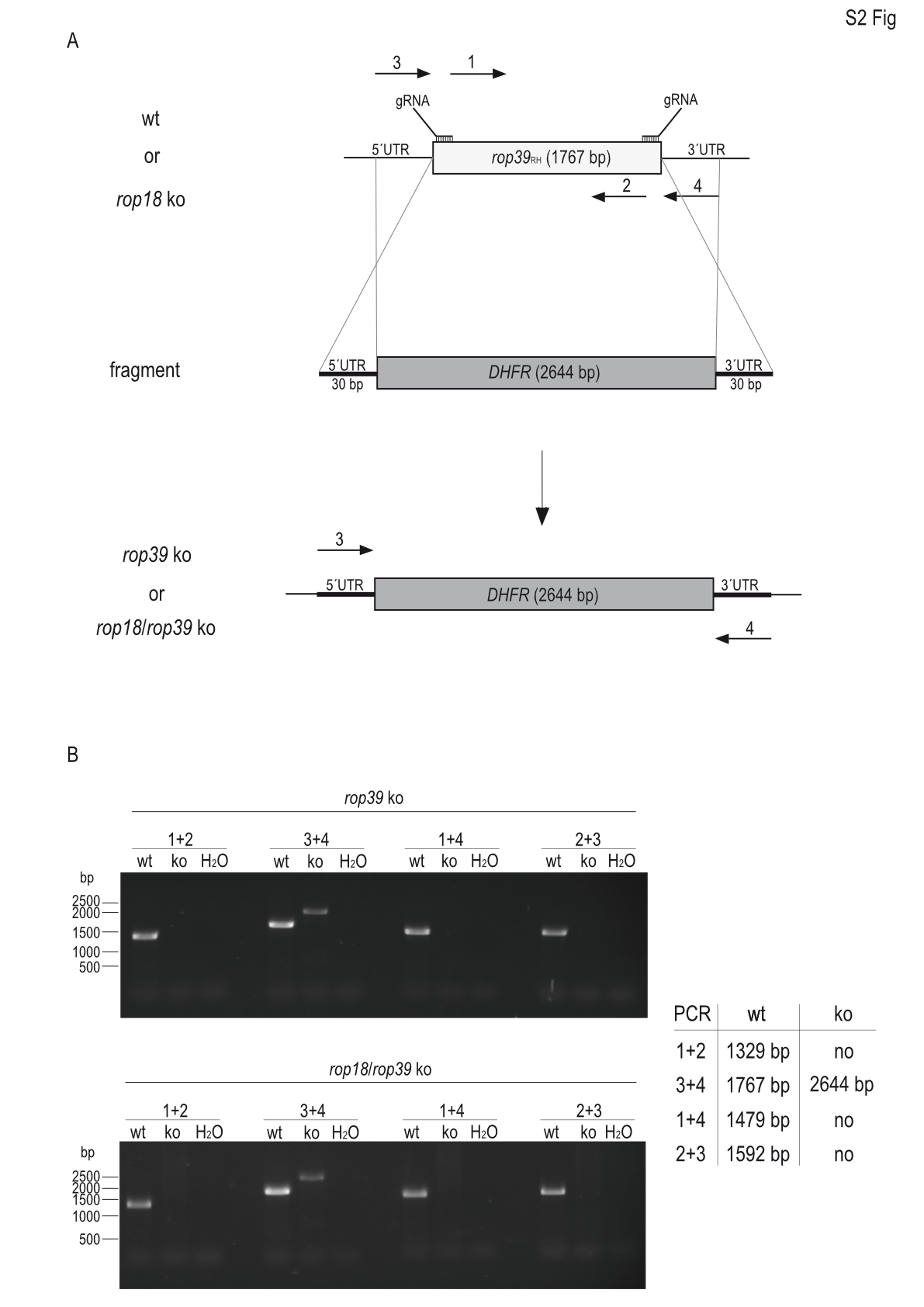

Supplement: S2 Fig — Generation of T. gondii RHΔrop39 and RHΔrop18/rop39 deletion mutants. A, Schematic representation of specific gRNA-mediated targeting of CRISPR/Cas9 to the endogenous rop39 locus and integration of the DHFR selection cassette carrying rop39 5`and 3`UTR homology regions. B, Diagnostic PCR of gDNA prepared from clonal lines demonstrates excision and replacement of the rop39 exon with the DHFR selection cassette. (TIFF) [file ppat.1011003.s002.tiff]

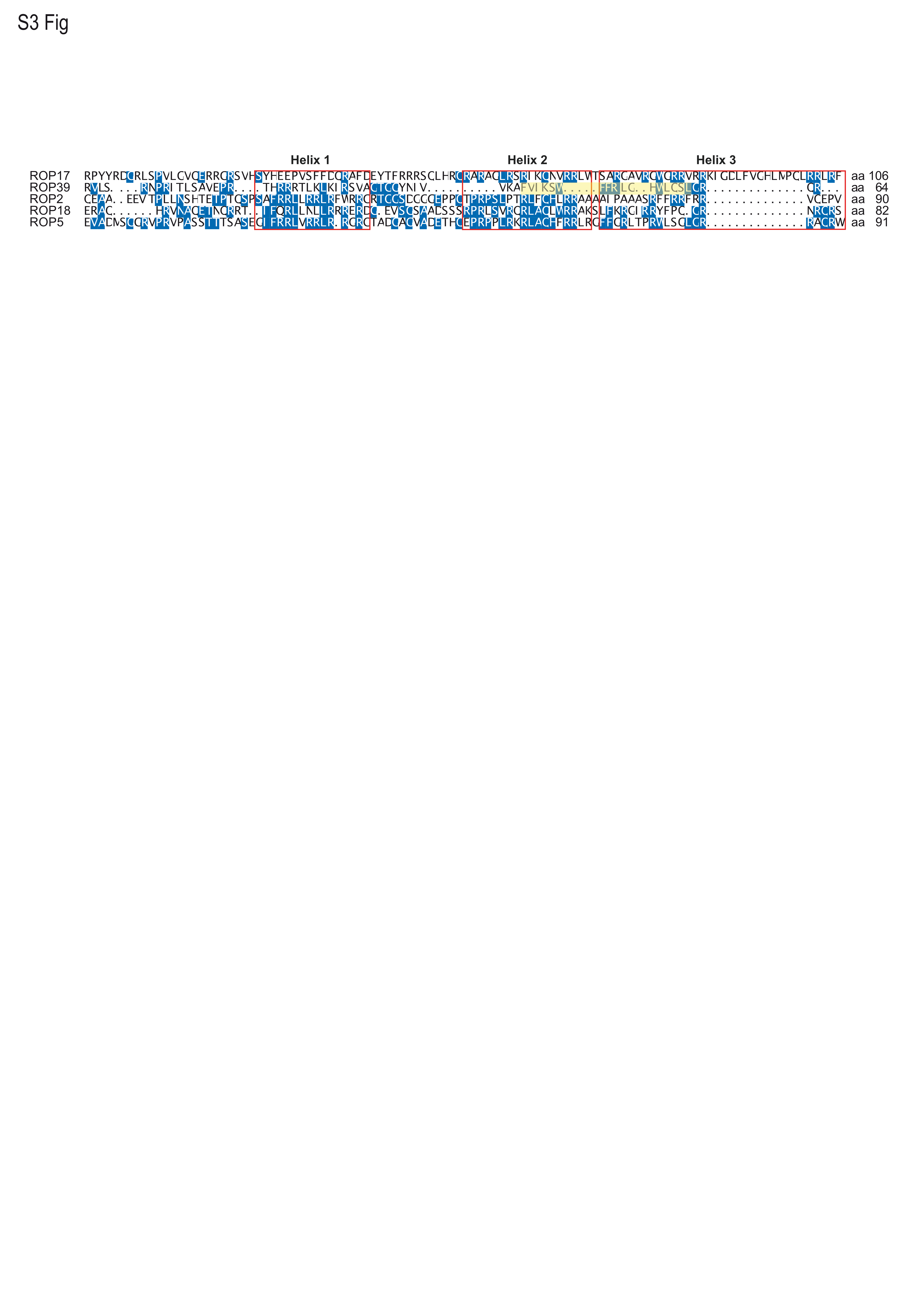

Supplement: S3 Fig — Multiple amino acid sequence alignment. Multiple amino acid alignment of the N-terminus of indicated rop genes from T. gondii GT-1. Letters in white, non conserved amino acids; letters in blue, conserved amino acids. Red boxes show amphipathic α-helices that mediate membrane targeting in case of ROP2, ROP5, ROP17 and ROP18. In case of ROP39, these sequences are highly degenerate but a single large amphipathic α-helix can be identified instead (yellow). (TIFF) [file ppat.1011003.s003.tiff]

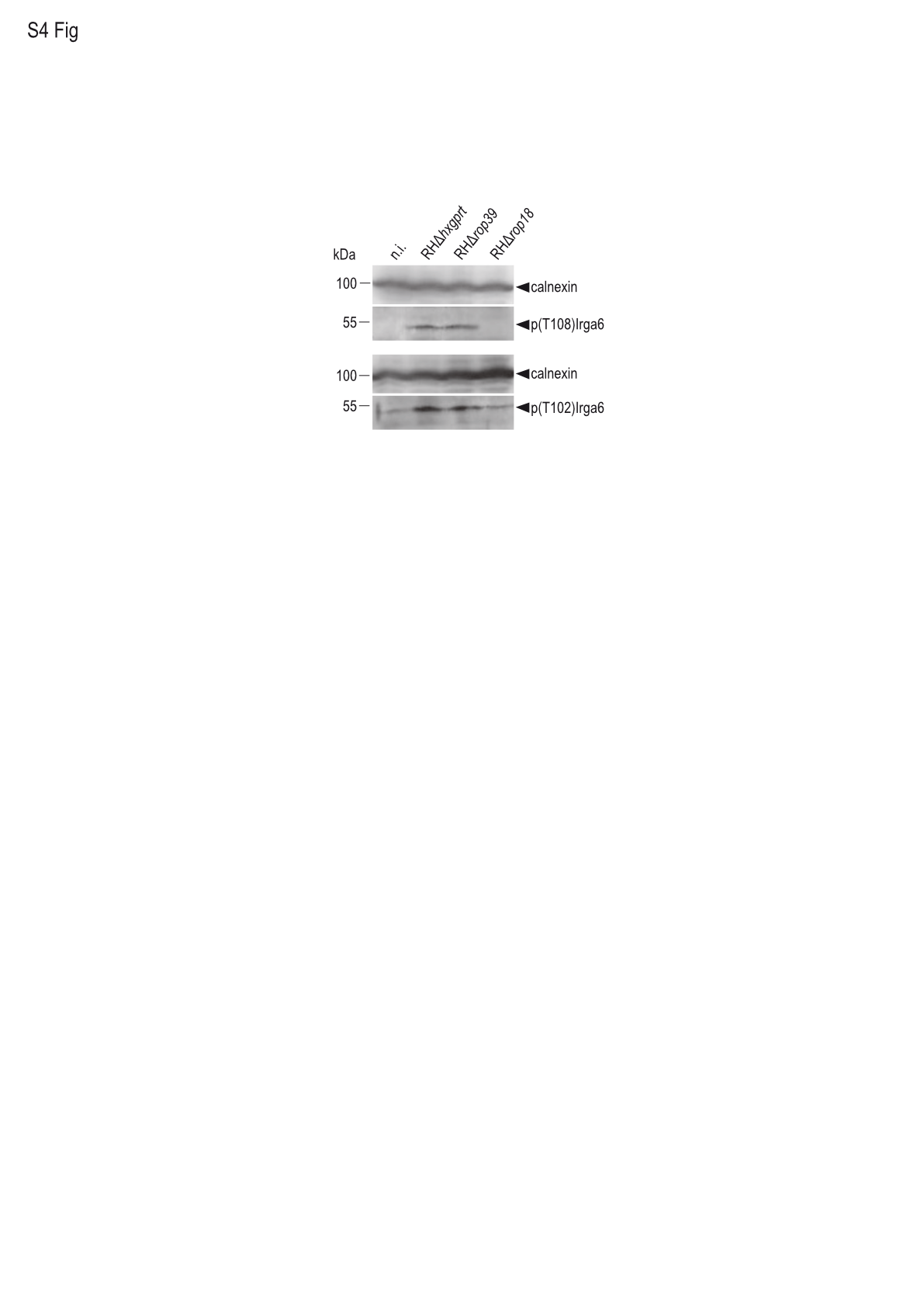

Supplement: S4 Fig — (T102)Irga6 and (T108)Irga6 are no targets of ROP39-mediated phosphorylation. Phosphorylation of Irga6 at threonine residues T102 ((T102)Irga6) or T108 ((T108)Irga6) is demonstrated upon infection with RHΔhxgprt or RHΔrop39 by Western blot using anti p(T102)Irga6- or p(T108)Irga6-specific antibodies. (TIFF) [file ppat.1011003.s004.tiff]

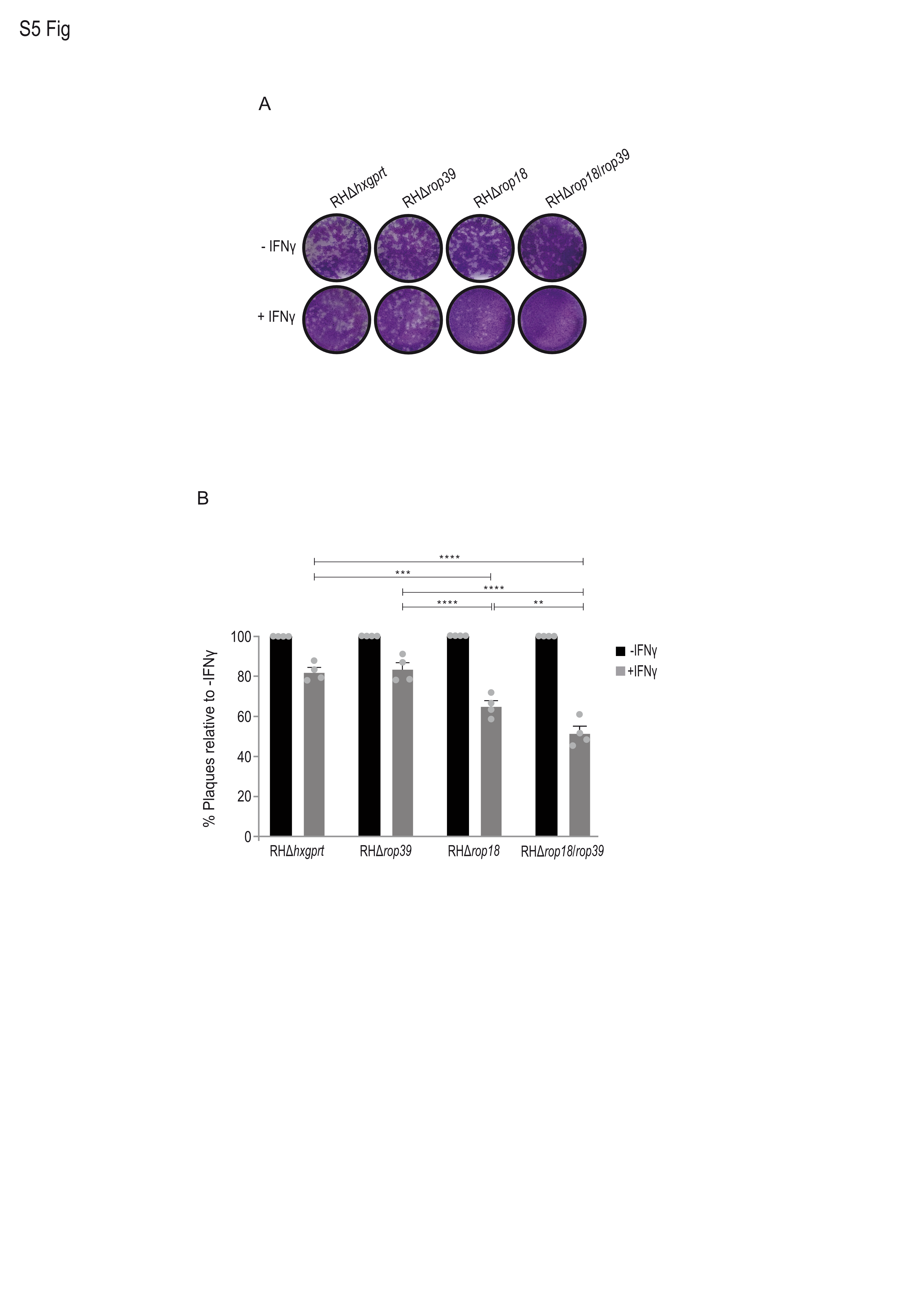

Supplement: S5 Fig — Plaque assay. Mouse embryonic fibroblasts grown in 6 well-plates in presence or absence of IFNγ (100 U/ml) for 24 h were infected with 200 freshly prepared T. gondii tachyzoites. A, At 7 d post infection, cell monolayers were stained with crystal violet. One representative experiment is depicted. B, The percentage of plaque number reduction for each T. gondii strain in presence of IFNγ (+IFNγ) in comparison to non-stimulated conditions (-IFNγ, set at 100%) is shown. Error bars indicate the mean and standard error of the mean (SEM) of four independent experiments. One-way analysis of variance (ANOVA) followed by Tukey´s multiple comparison was used to test differences between groups; ****p < 0.0001, ***p < 0.001; **p < 0.01. (TIFF) [file ppat.1011003.s005.tiff]

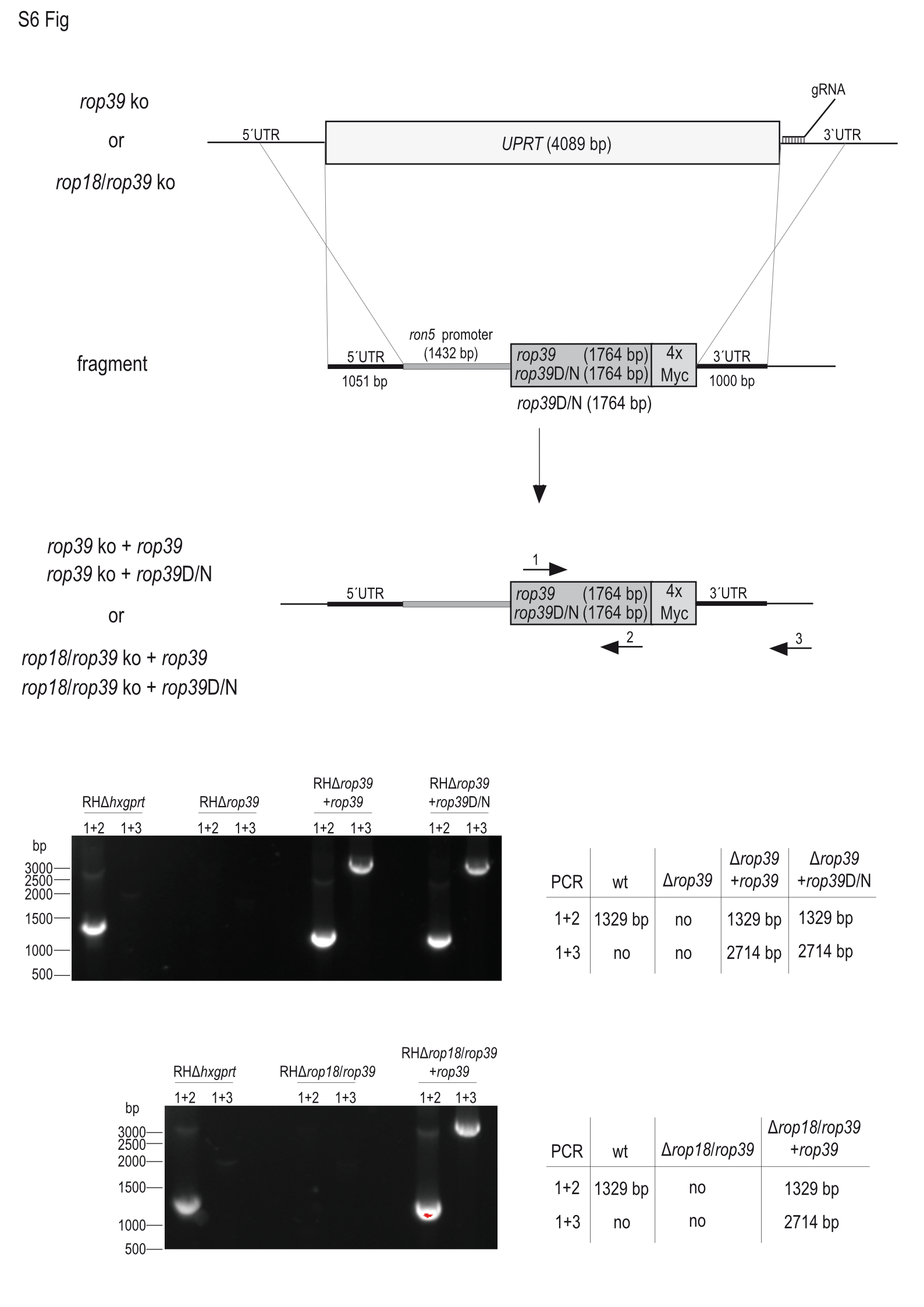

Supplement: S6 Fig — Generation of T. gondii RHΔrop39+rop39 and RHΔrop18/rop39+rop39. Schematic representation of CRISPR/Cas9 gRNA-mediated integration of C-terminally Myc-tagged rop39 including the ron5 promoter at the endogenous UPRT locus. (TIFF) [file ppat.1011003.s006.tiff]

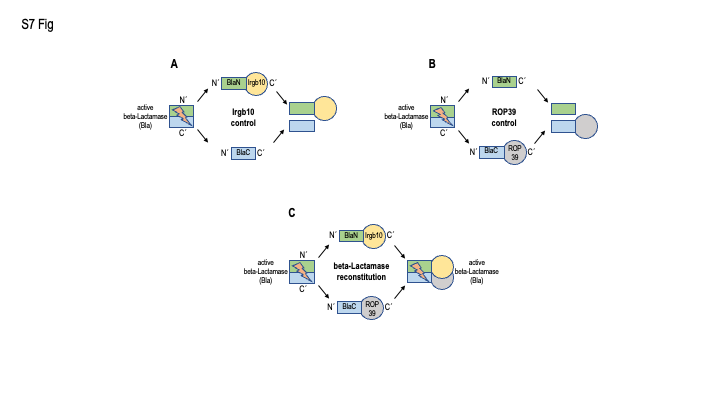

Supplement: S7 Fig — Exemplary schematic representation of a PCA reaction. Irgb10 and T. gondii type I ROP39 were fused to N-terminal (BlaN) or C-terminal (BlaC) fragments of the reporter protein TEM-1 β-lactamase respectively. A, Control reaction containing Irgb10 fused to the N-terminal fragment of β-lactamase (BlaN-Irgb10) and an empty plasmid containing only the C-terminal β-lactamase fragment. No restoration of β-lactamase activity is expected. B, Control reaction containing ROP39 fused to the C-terminal fragment of β-lactamase (BlaC-ROP39) and an empty plasmid containing only the N-terminal β-lactamase fragment. No restoration of β-lactamase activity is expected. C, Reaction containing Irgb10 fused to the N-terminal fragment of β-lactamase (BlaN-Irgb10) and ROP39 fused to the C-terminal fragment of β-lactamase (BlaC-ROP39). Upon Irgb10:ROP39-interaction, β-lactamase activity is restored. (TIFF) [file ppat.1011003.s007.tiff]

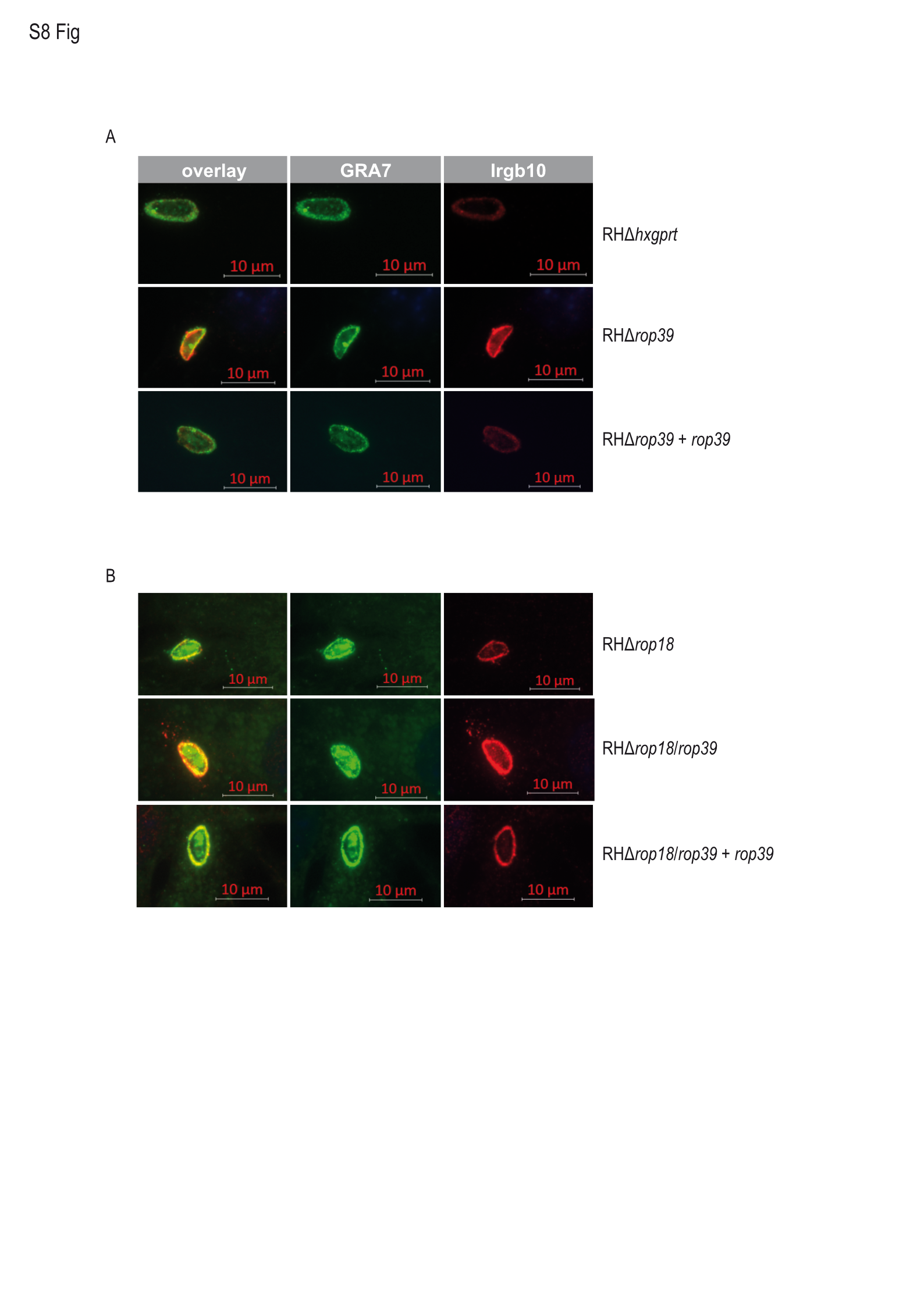

Supplement: S8 Fig — Representative fluorescent images of Irgb10-positive vacuoles. A, B, Mouse embryonic fibroblasts have been stimulated with 200 U/ml IFNγ for 24 h and infected with indicated T. gondii strains at MOI 5. After 2 h, cells were prepared for immunofluorescence analysis as described in Materials and methods. Irgb10 in red (right hand panels), GRA7 in green (middle panels) and overlay (left hand panels) are shown. Pictures for Irgb10-positive vacuoles were taken at the same exposure time. Scale bars, 10 μm. (TIFF) [file ppat.1011003.s008.tiff]

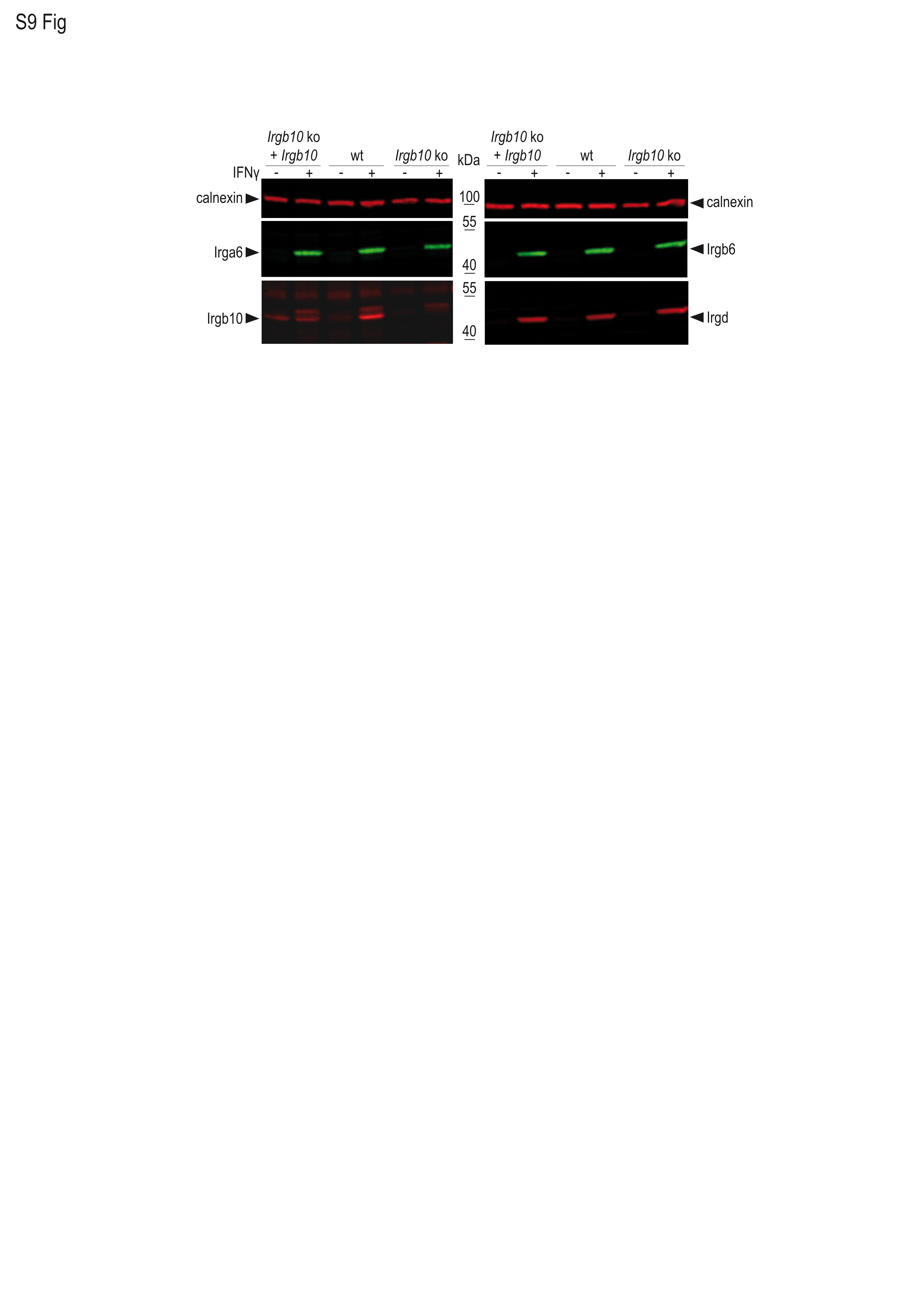

Supplement: S9 Fig — IRG protein expression levels are unaffected in Irgb10 ko cells. Western blot of detergent lysates from BL/6 wild-type (wt), Irgb10 knockout (ko) and Irgb10 complemented ko cells stimulated for 24 h with 200 U/ml IFNγ. The signal representing Irgb10 in wt and complemented cells is lost in Irgb10 ko cells. Expression levels of all other Immunity-Related GTPases (IRG proteins) are unchanged in ko cells compared with wt and complemented cells. Calnexin serves as loading control. (TIFF) [file ppat.1011003.s009.tiff]

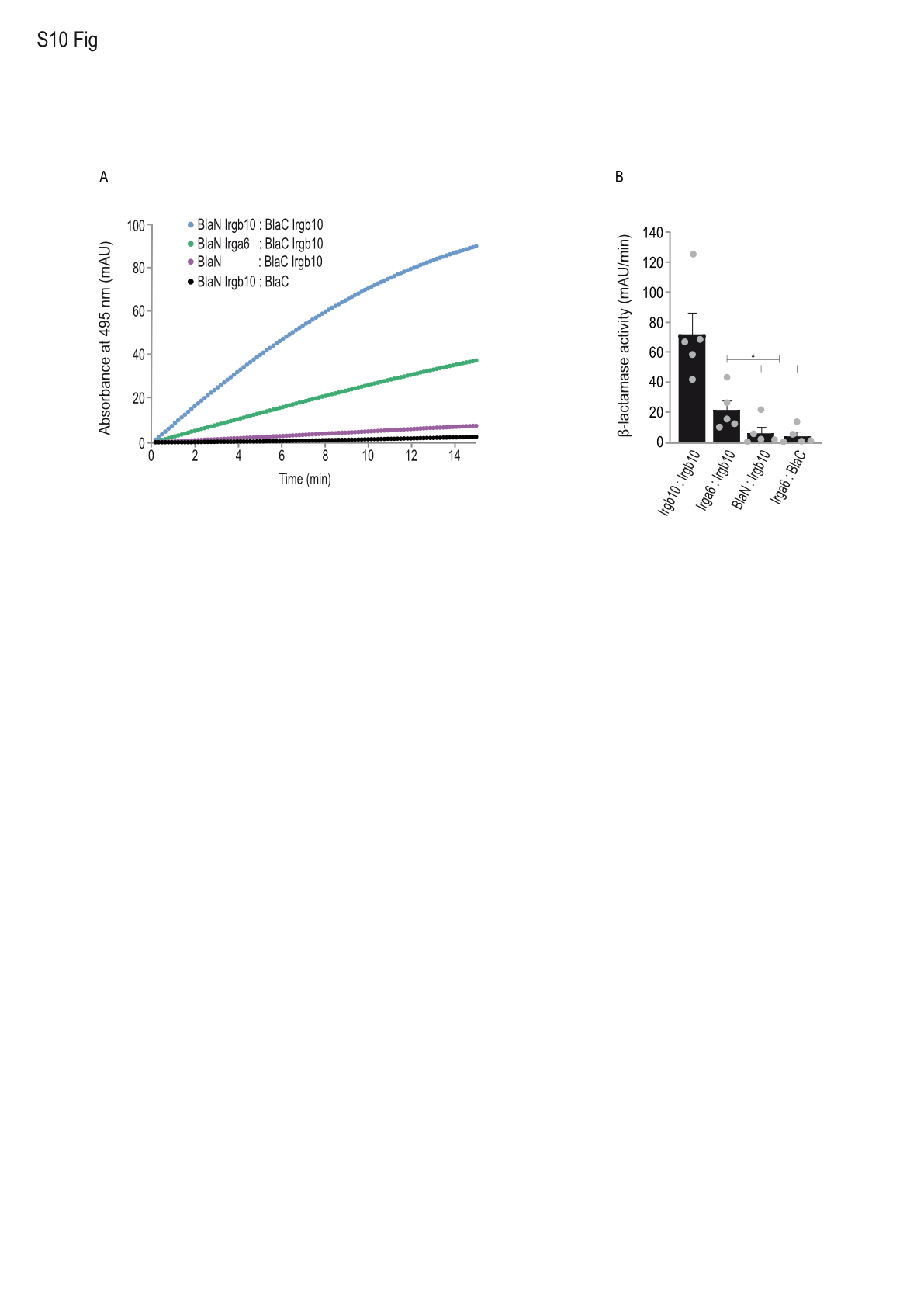

Supplement: S10 Fig — Irgb10 and Irga6 form heterodimers. Protein-fragment complementation assay. Proteins were fused to N-terminal (BlaN) or C-terminal (BlaC) fragments of the reporter protein TEM-1 β-lactamase (Bla). The increase in absorbance measured at 495 nm indicates restoration of β-lactamase activity after protein:protein-interaction. A, The kinetic of the β-lactamase reaction is shown for one representative experiment. B, Heterodimerisation of Irgb10 and Irga6. Error bars indicate the mean and standard deviation of three independent experiments. One-way analysis of variance (ANOVA) followed by Tukey’s multiple comparison was used to test differences between groups; *p < 0.05. (TIFF) [file ppat.1011003.s010.tiff]

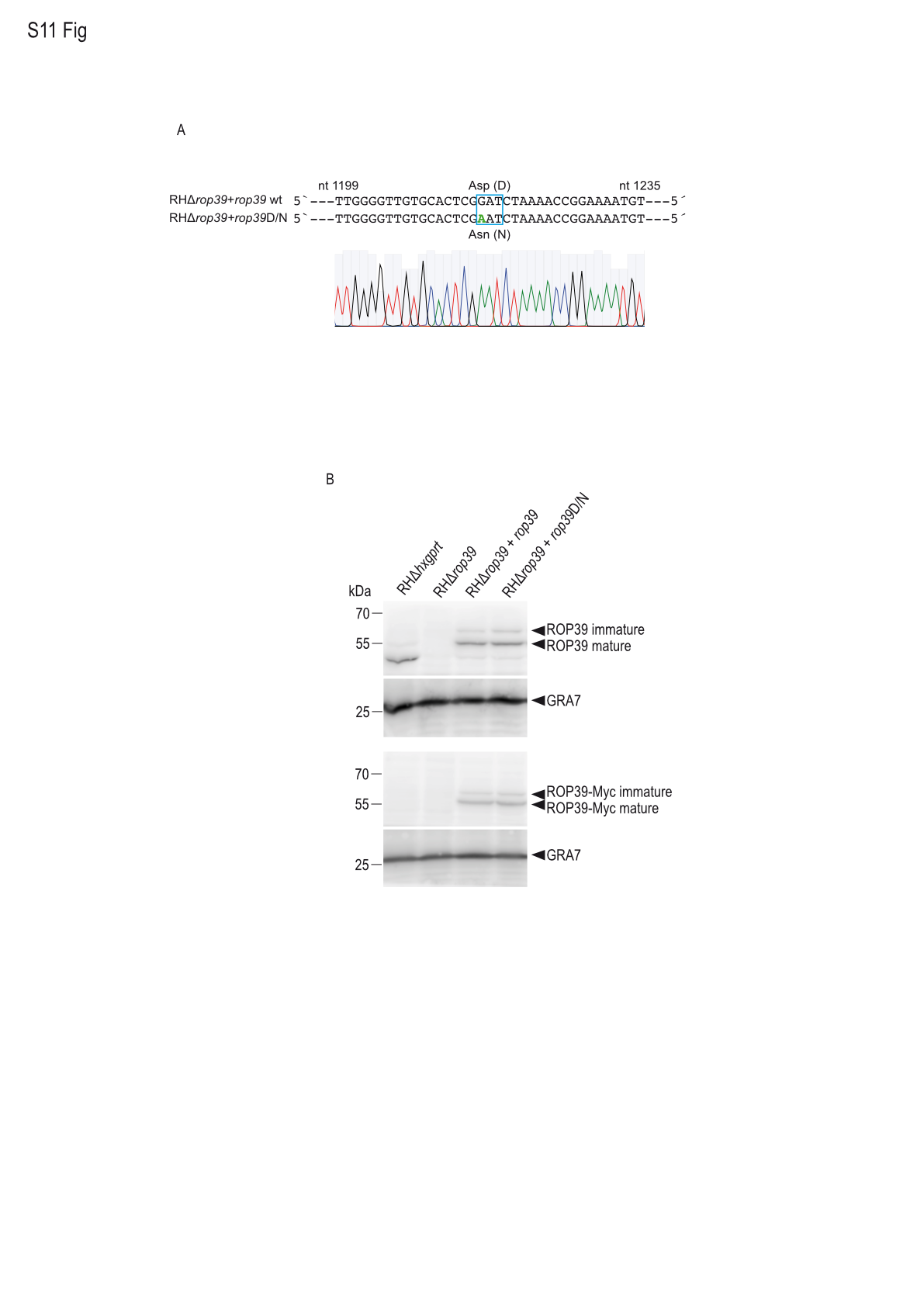

Supplement: S11 Fig — Confirmation of T. gondii RHΔrop39+rop39D/N. A, Complementation of T. gondii RHΔrop39 with a kinase dead mutant of ROP39 (ROP39D/N) was confirmed by Sanger sequencing of a rop39-specific PCR product. The nucleotide exchange from G to A (green) at nt position 1216 leading to an amino acid exchange of the key catalytic aspartate (Asp, D) at position 406 to asparagine (Asn, N) (blue box, upper panel) and the electropherogram (lower panel) is depicted. B, Complementation of RHΔrop39 with the kinase dead version of ROP39 (RHΔrop39+rop39D/N) is demonstrated in comparison to RHΔhxgprt, RHΔrop39 and RHΔrop39+rop39 by Western blot using a ROP39-specific peptide antiserum (upper panel) or anti-Myc antibody (lower panel). (TIFF) [file ppat.1011003.s011.tiff]

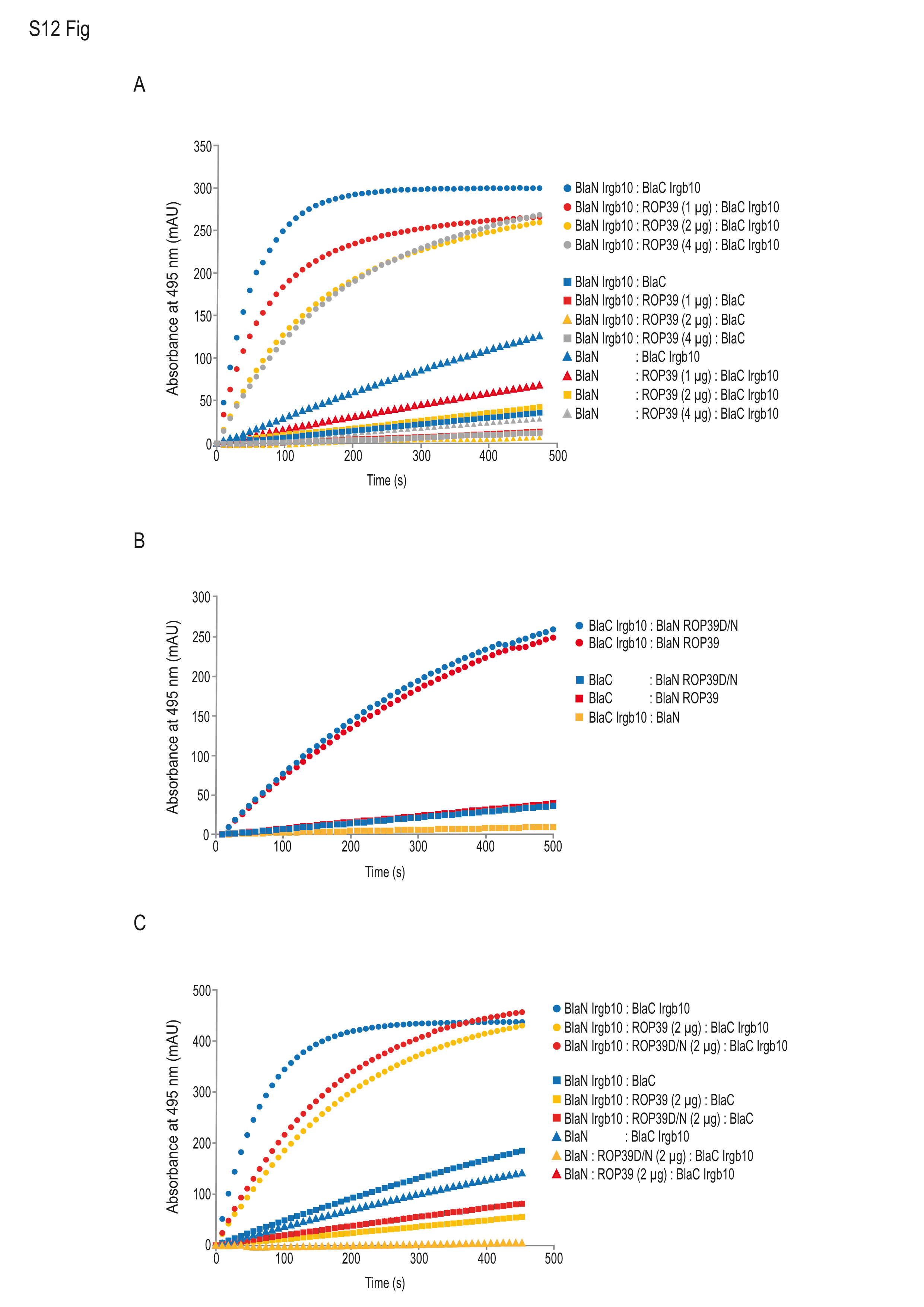

Supplement: S12 Fig — ROP39 inhibits homodimerisation of Irgb10. Protein-fragment complementation assay. Proteins were fused to N-terminal (BlaN) or C-terminal (BlaC) fragments of the reporter protein TEM-1 β-lactamase (Bla). The increase in absorbance measured at 495 nm indicates restoration of β-lactamase activity after protein:protein-interaction. A, Homodimerisation of Irgb10 is inhibited in the presence of ROP39. B, ROP39 wt and the kinase dead version of ROP39 (ROP39D/N) bind to Irgb10. C, Homodimerisation of Irgb10 is inhibited in the presence of ROP39 wt and the kinase dead version of ROP39 (ROP39D/N). A, B, C The kinetic of the β-lactamase reaction is shown for one representative experiment. (TIFF) [file ppat.1011003.s012.tiff]

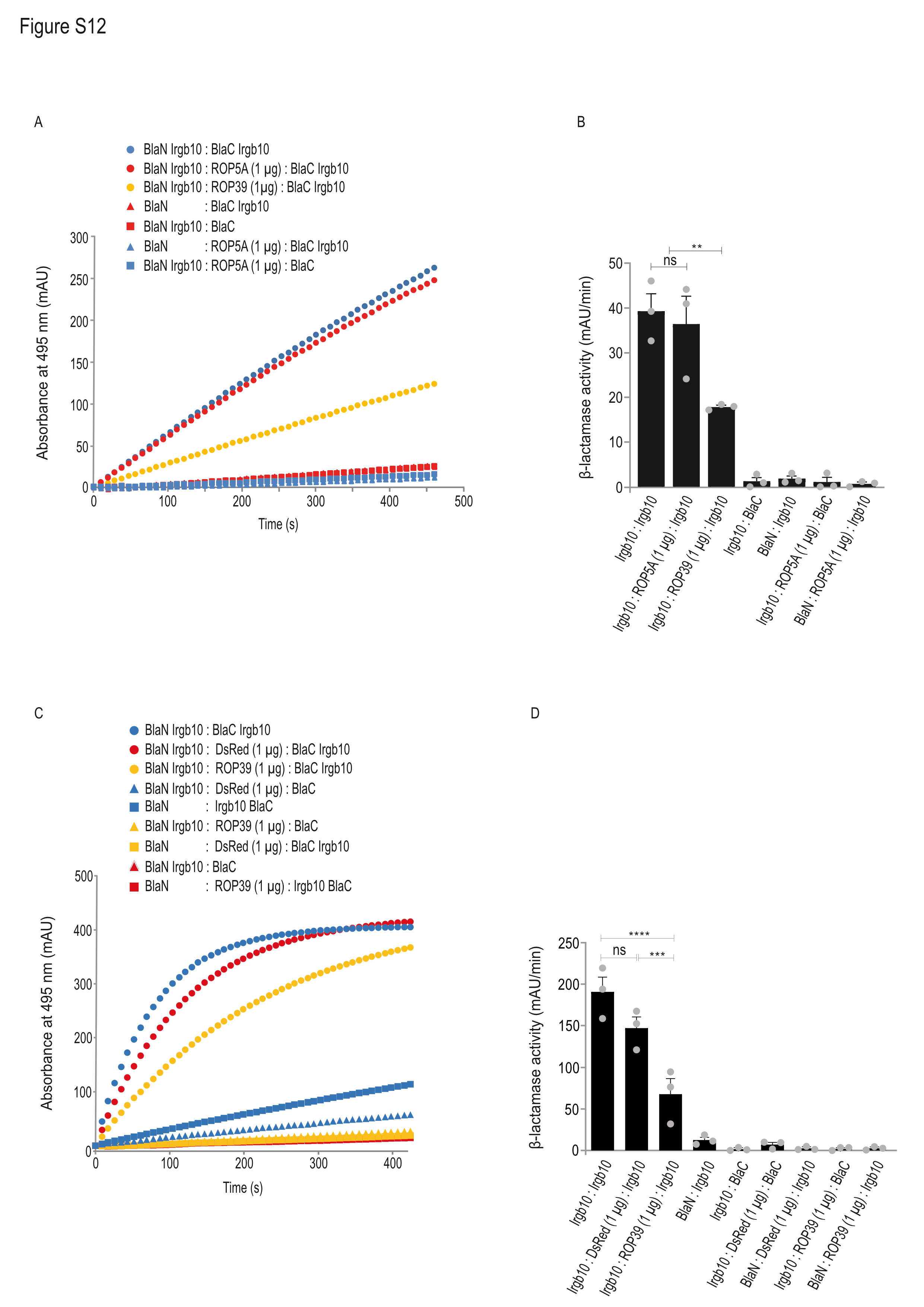

Supplement: S13 Fig — PCA inert protein controls. Protein-fragment complementation assay. Proteins were fused to N-terminal (BlaN) or C-terminal (BlaC) fragments of the reporter protein TEM-1 β-lactamase (Bla). The increase in absorbance measured at 495 nm indicates restoration of β-lactamase activity after protein:protein-interaction. A, Homodimerisation of Irgb10 is not inhibited in the presence of ROP5A as an inert protein control. B, The kinetic of the β-lactamase reaction is shown for one representative experiment. C, Homodimerisation of Irgb10 is not inhibited in the presence of DsRed as an inert protein control. D, The kinetic of the β-lactamase reaction is shown for one representative experiment. (TIFF) [file ppat.1011003.s013.tiff]

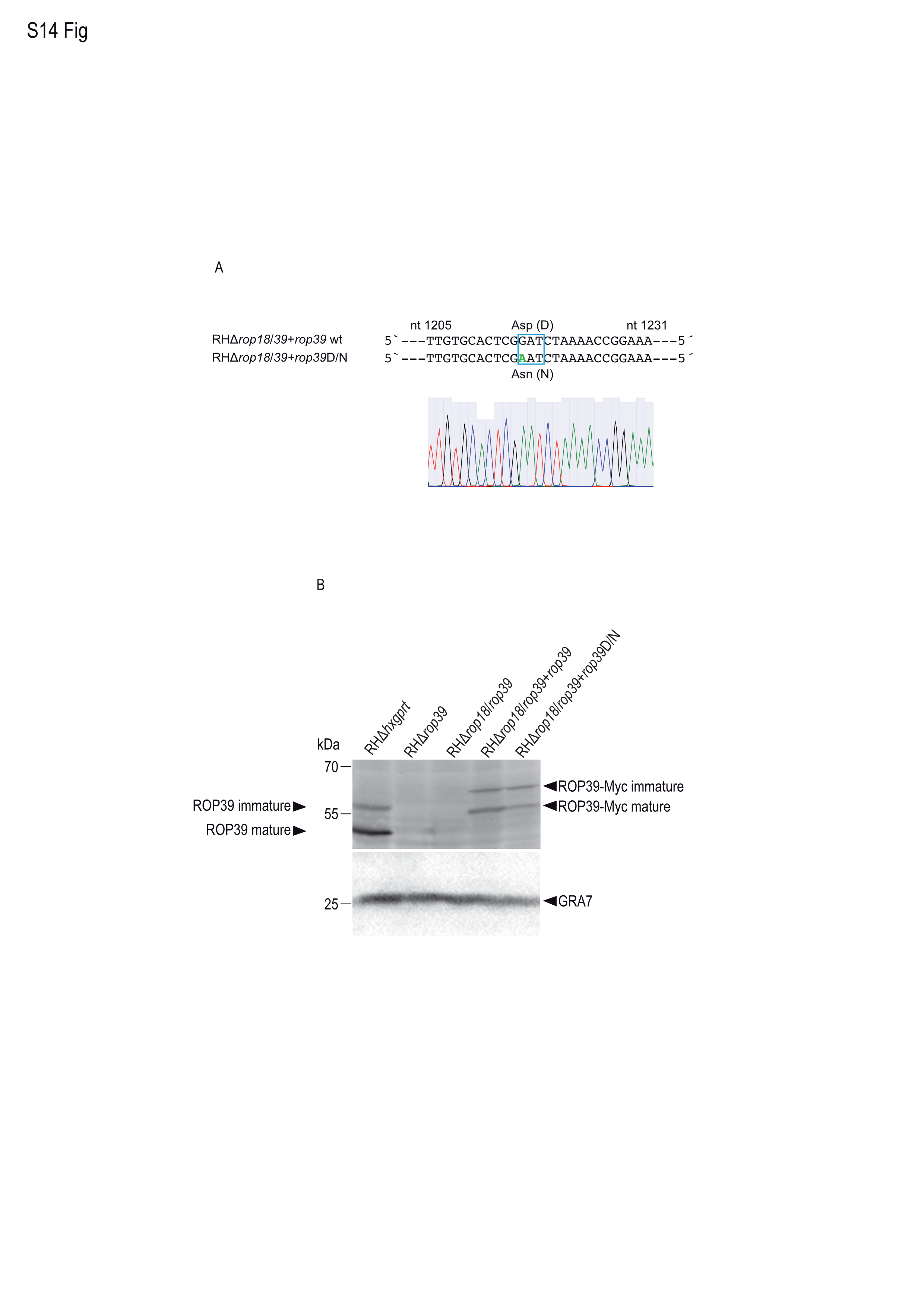

Supplement: S14 Fig — Confirmation of T. gondii RHΔrop18/rop39+rop39D/N. A, Complementation of T. gondii RHΔrop18/rop39 with a kinase dead mutant of ROP39 (ROP39D/N) was confirmed by Sanger sequencing of a rop39-specific PCR product. The nucleotide exchange from G to A (green) at nt position 1216 leading to an amino acid exchange of the key catalytic aspartate (Asp, D) at position 406 to asparagine (Asn, N) (blue box, upper panel) and the electropherogram (lower panel) is depicted. B, Complementation of RHΔrop18/rop39 with the kinase dead version of ROP39 (RHΔrop18/rop39+rop39D/N) is demonstrated in comparison to RHΔhxgprt, RHΔrop39, RHΔrop18/rop39 and RHΔrop18/rop39+rop39 by Western blot using a ROP39-specific peptide antiserum. (TIFF) [file ppat.1011003.s014.tiff]

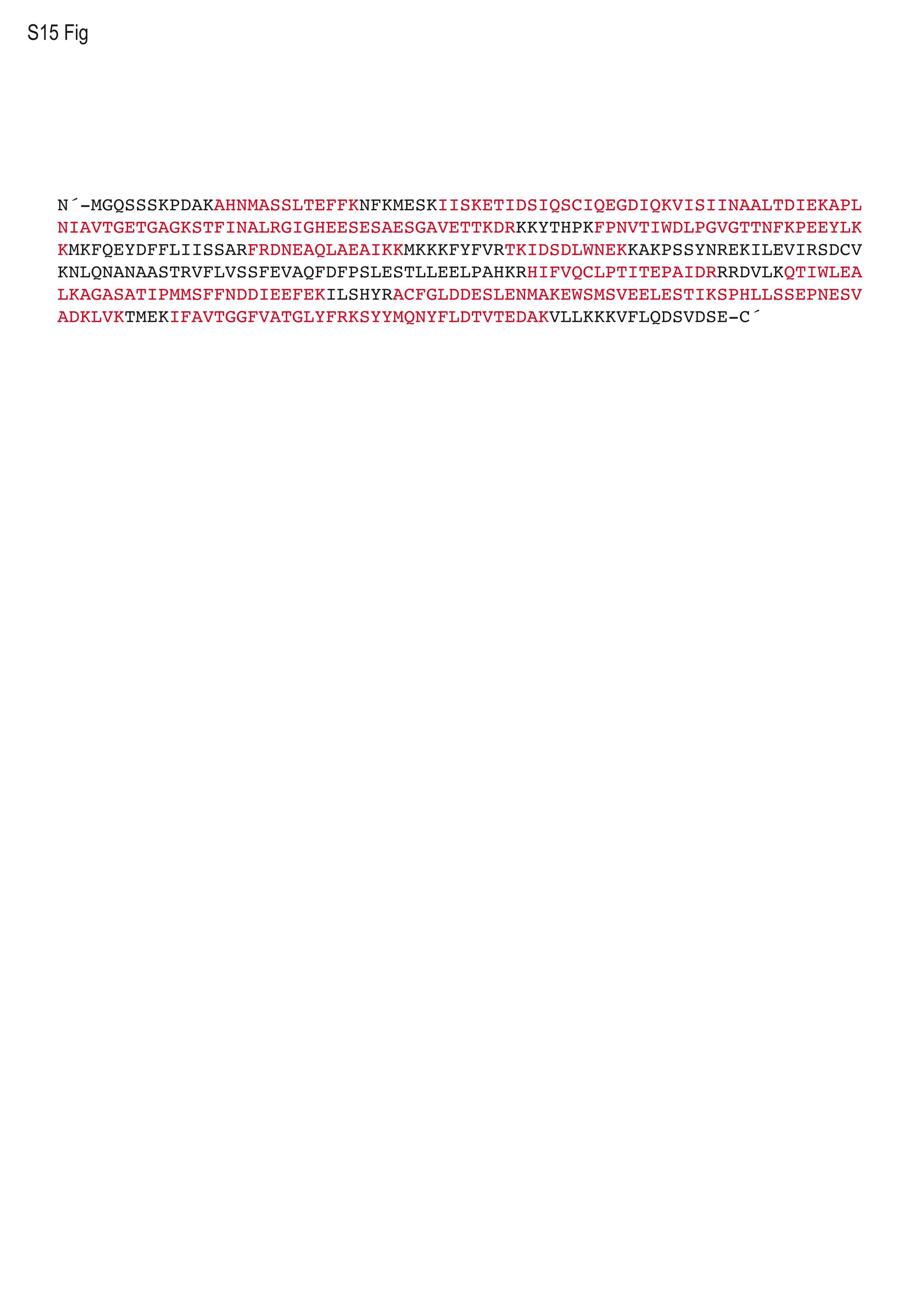

Supplement: S15 Fig — Tryptic peptides of immunoprecipitated Irgb10 from IFNγ-induced and RHΔhxgprt-inefected MEFs using an Irgb10-specific antiserum. Identified peptides corresponding to Irgb10 are marked in red (sequence coverage 65,2%). No phosphosite could be revealed in this MS analysis. (TIFF) [file ppat.1011003.s015.tiff]
