# Supplementary material for: ROP39 is an Irgb10-specific parasite effector that modulates acute Toxoplasma gondii virulence
Source: PLoS Pathog. 2023 Jan 5;19(1):e1011003. doi: 10.1371/journal.ppat.1011003 (PMC9848475; doi:10.1371/journal.ppat.1011003)
Supplement: S1 Table — (PDF) [file ppat.1011003.s016.pdf]

S1 Table. Reagents and resources.

Name	Source
Bacteria	
DH5α	Zymo Research
Invitrogen™ One Shot™ PIR1 Chemically Competent *E. coli*	Thermo Fisher Scientific
Rosetta™ (DE3) Competent Cells	Novagen
Cell lines	
Mouse embryonic fibroblasts (MEF)	University Medical Center Freiburg
Human foreskin fibroblasts (HFF)	ATCC
HEK (Human Embryo Kidney) 293 T (SV40 large T antigen) cells	ATCC
Yeast	
Saccharomyces Cerevisiae strain PJ69-4α	[1]
Mouse strains	
C57BL/6 mice	Janvier Labs
Irgm1/Irgm3$^{-/-}$ mice	University Medical Center Freiburg
Chemicals	
GSTrap FF Glutathione Sepharose affinity column	Merck
3-AT (3-amino-1,2,4-triazole)	Merck
Nitrocefin	Abcam
5-Fluoro-2'-deoxyuridine	Sigma
Pyrimethamine	Sigma
Zeocin	Invivogen
Invitrogen™ Lipofectamine™ 3000	Thermo Fisher Scientific
Protease inhibitor cocktail tablets	Roche
Passive Lysis Buffer (5x)	Promega
Gibson Assembly Cloning Kit	New England Biolabs
Quick-DNA Miniprep Kit	Zymo Research
Software	
GraphPad Prism 8	GraphPad software
ApE-A plasmid editor	[2]
PSIPRED 4.0	[3]
MEGA-X	[4]
HeliQuest	[5]
Adobe illustrator	Adobe Inc.

1. Peterson TA, Stamnes MA, Piper RC (2018) A Yeast 2-Hybrid Screen in Batch to Compare Protein Interactions. Journal of visualized experiments : JoVE (136).
2. Davis MW, Jorgensen EM (2022) ApE, A Plasmid Editor: A Freely Available DNA Manipulation and Visualization Program. Front. Bioinform. 2: 5. Available: https://www.frontiersin.org/articles/10.3389/fbinf.2022.818619/full.
3. Buchan DWA, Jones DT (2019) The PSIPRED Protein Analysis Workbench: 20 years on. Nucleic acids research 47 (W1): W402-W407.
4. Kumar S, Stecher G, Li M, Knyaz C, Tamura K (2018) MEGA X: Molecular Evolutionary Genetics Analysis across Computing Platforms. Molecular biology and evolution 35 (6): 1547–1549.
5. Gautier R, Douguet D, Antonny B, Drin G (2008) HELIQUEST: a web server to screen sequences with specific alpha-helical properties. Bioinformatics (Oxford, England) 24 (18): 2101–2102. Available: https://pubmed.ncbi.nlm.nih.gov/18662927/.
