# Supplementary material for: ROP39 is an Irgb10-specific parasite effector that modulates acute Toxoplasma gondii virulence
Source: PLoS Pathog. 2023 Jan 5;19(1):e1011003. doi: 10.1371/journal.ppat.1011003 (PMC9848475; doi:10.1371/journal.ppat.1011003)
Supplement: S2 Table — (PDF) [file ppat.1011003.s017.pdf]

S2 Table. *T. gondii* strains used in this study.

Name	Source
T. gondii RHΔ*hxgprt*	[1]
T. gondii RHΔ*hxgprt*/Δ*rop18* (RHΔ*rop18*)	[2]
T. gondii RHΔ*hxgprt*/Δ*rop5* (RHΔ*rop5*)	[3]
T. gondii RHΔ*hxgprt*/Δ*rop39* (RHΔ*rop39*)	This study
T. gondii RHΔ*hxgprt*/Δ*rop18*/Δ*rop39* (RHΔ*rop18*/*rop39*)	This study
T. gondii RHΔ*hxgprt*/Δ*rop39*+*rop39* (RHΔ*rop39*+*rop39*)	This study
T. gondii RHΔ*hxgprt*/Δ*rop18*/Δ*rop39*+*rop39* (RHΔ*rop18*/*rop39*+*rop39*)	This study
T. gondii : RHΔ*hxgprt*/Δ*rop39*+*rop39*D/N (RHΔ*rop39*+*rop39*D/N)	This study
T. gondii RHΔ*hxgprt*/Δ*rop18*/Δ*rop39*+*rop39*D/N (RHΔ*rop18*/*rop39*+*rop39*D/N)	This study

1. Roos DS, Donald RGK, Morrissette NS, Moulton ALC (1994) Chapter 3: Molecular Tools for Genetic Dissection of the Protozoan Parasite Toxoplasma gondii. In: Russell DG, editor. Microbes as tools for cell biology. San Diego, Calif., London: Academic Press. pp. 27–63.
2. Behnke MS, Fentress SJ, Mashayekhi M, Li LX, Taylor GA et al. (2012) The polymorphic pseudokinase ROP5 controls virulence in Toxoplasma gondii by regulating the active kinase ROP18. PLoS pathogens 8 (11): e1002992.
3. Behnke MS, Khan A, Wootton JC, Dubey JP, Tang K et al. (2011) Virulence differences in Toxoplasma mediated by amplification of a family of polymorphic pseudokinases. Proceedings of the National Academy of Sciences of the United States of America 108 (23): 9631–9636.
