# Supplementary material for: ROP39 is an Irgb10-specific parasite effector that modulates acute Toxoplasma gondii virulence
Source: PLoS Pathog. 2023 Jan 5;19(1):e1011003. doi: 10.1371/journal.ppat.1011003 (PMC9848475; doi:10.1371/journal.ppat.1011003)
Supplement: S3 Table — (PDF) [file ppat.1011003.s018.pdf]

S3 Table. Plasmids and constructs used in this study.

Name	Experiment	Source
pGAD-T7-*rop39*	Y2H	This study
pGBD-T7-*rop39*	Y2H	This study
pGAD-T7-*rop5A*	Y2H	[1]
pGBD-T7-*rop5A*	Y2H	[1]
pGAD-T7-*rop5B*	Y2H	[1]
pGBD-T7-*rop5B*	Y2H	[1]
pGAD-T7-*rop5C*	Y2H	[1]
pGBD-T7-*rop5C*	Y2H	[1]
pGAD-T7-*Irga6*	Y2H	[1]
pGBD-T7-*Irga6*	Y2H	[1]
pGAD-T7-*Irgb10*	Y2H	This study
pGBD-T7-*Irgb10*	Y2H	This study
pGAD-T7-*Irgb6*	Y2H	This study
pGBD-T7-*Irgb6*	Y2H	This study
pGAD-T7-*Irgd*	Y2H	This study
pGBD-T7-*Irgd*	Y2H	This study
pGEX-4T-*rop39*	pull down	This study
pU6-*rop39*	generation of *T. gondii rop39* and *rop18*/*rop39* ko strains	This study
pUPRT-RON5-*rop39*-Myc	complementation of *T. gondii* ko strains	This study
pUPRT-RON5-*rop39D/N*-Myc	complementation of *T. gondii* ko strains	This study
pU6-UPRT	complementation of *T. gondii* ko strains	[2]
BlaN	PCA	[3]
BlaC	PCA	[3]
BlaN-*Irgb10*	PCA	This study
BlaN-*Irga6*	PCA	This study
BlaN-*Irgb6*	PCA	This study
BlaC-*Irgb10*	PCA	This study
BlaC-*rop39*	PCA	This study
BlaC-*rop39*D/N	PCA	This study
puc2CL6IPwo	PCA	[4]
puc2CL6IPwo-*rop39*	PCA	This study
puc2CL6IPwo-*rop39*D/N	PCA	This study
pdsRed-c3	PCA	TaKaRa Bio Inc.

1. Murillo-León M, Müller UB, Zimmermann I, Singh S, Widdershooven P et al. (2019) Molecular mechanism for the control of virulent Toxoplasma gondii infections in wild-derived mice. Nature communications 10 (1): 1233.
2. Guérin A, Corrales RM, Parker ML, Lamarque MH, Jacot D et al. (2017) Efficient invasion by Toxoplasma depends on the subversion of host protein networks. Nat Microbiol 2 (10): 1358–1366.
3. Schnee M, Ruzsics Z, Bubeck A, Koszinowski UH (2006) Common and specific properties of herpesvirus UL34/UL31 protein family members revealed by protein complementation assay. Journal of virology 80 (23): 11658–11666.
4. Matschulla T, Berry R, Gerke C, Döring M, Busch J et al. (2017) A highly conserved sequence of the viral TAP inhibitor ICP47 is required for freezing of the peptide transport cycle. Sci Rep 7 (1): 2933. Available: https://www.nature.com/articles/s41598-017-02994-5#citeas.
