# Supplementary material for: ROP39 is an Irgb10-specific parasite effector that modulates acute Toxoplasma gondii virulence
Source: PLoS Pathog. 2023 Jan 5;19(1):e1011003. doi: 10.1371/journal.ppat.1011003 (PMC9848475; doi:10.1371/journal.ppat.1011003)
Supplement: S4 Table — (PDF) [file ppat.1011003.s019.pdf]

S4 Table. Oligonucleotides and primers used in this study.

**Name**	**sequence (5'-3')**
UPRT plasmid sequencing-fw	CAGAAGACTTTTCCTTTGAGTCT
UPRT plasmid sequencing-bw	GGACACAGGGGTCTCTGGCACCC
UPRT-3'homology diagnostic PCR-fw	GGATCCGCACGAAATCATCTTCGCA
UPRT-3'homology diagnostic PCR-bw	CTACCTTTCCTACAGGATGCCTTGCAATG
DHFR diagnostic PCR-fw	TATGGCACTGGAATCCGTTCATTGTCAGT
DHFR diagnostic PCR-bw	GCAAAACAGCCTCAACATAACGCTCCAA
ROP39 internal-fw	ACAACCTCGGCTTTAAACGTGAACC
ROP39 internal-bw	CCACCCGAGGATTATCACAGCCTAT
UPRT-3' UTR-bw sequencing	GGAGGAAAGTAAAACTTCAGTT
rop39-UPRT-pRON5-gibson-fw	CCTTTGAGTCTTCAGAGGCGCCTTAGCCAAAATGAGCAAACCTTTTTCCCACAGG
rop39-UPRT-pRON5-gibson-bw	CTCTTCTGAGATGAGTTTTTGTTCGATATCAACAATTGATTCCCGAAGAGGCAAAA
rop39 ko-25 gRNA	GTAAATGGGGATGTCAAGTTGTGCGGGTATCGTGTACAGGGTTTTAGAGCTAGAAATAGC
rop39 ko-1717-rev comp-gRNA	GCTATTTCTAGCTCTAAAACAAACGAGGCAAATCGGTCACAACTTGACATCCCCATTTAC
rop39-DHFR-homology	GGTATGGCACTGGAATCCGTTCATTGTCAGTGCGGCCGCTCTAGAACTAG
rop39-DHFR-homology-rev comp	GGGAGGCTGGGCCCGTCCCCAAATGACGACGCGGAAGATCCGATCTTGC
pU6-sequencing-fw	CCGTCGACACACGCGCCACATGGG
pU6-sequencing-bw	CAGGAAACAGCTATGACCATG
Irgb10 Y2H-NdeI-fw	CCCCCCCATATGGGTCAGTCTTCTTCTAAACCC
Irgb10 Y2H-BamHI-bw	CCCCCCGGATCCTTACTCAGAGTCCACACTGTCCTG
Y2H-Tg*rop39*-NdeI-fw	CCCCCCCATATGCTCCTTGATCGCGTTCTTTCCCG
Y2H-Tg*rop39*-EcoRI-bw	CCCCCCGAATTCTTAAACAATTGATTCCCGAAGAGGCAAA
Y2H-*Irgd*-EcoRI-fw	CCCCCCGAATTCATGGATCAGTTCATCTCAGCCTT
Y2H-*Irgd*-BamHI-bw	CCCCCCGGATCCCTAGGCAACATTTACTGTTTCTATCTTT
Irgb6-Y2H-fw	CCCCCCCATATGGCTTGGGCCTCCAGC
Irgb6-Y2H-bw	CCCCCCGGATCCTTACTCAAGCTTCCCAGTACTCGGG
rop39-D/N-fw	TGTGCACTCGAATCTAAAACCGG
rop39-D/N-bw	CCGGTTTTAGATTCGAGTGCACA
PCA-*Irgb10*-NheI-fw	CCCCCCGCTAGCATGGGTCAGTCTTCTTCTAAACCCG
PCA-*Irgb10*-NotI-bw	CCCCCCGCGGCCGCTTACTCAGAGTCCACACTGTCCTG
PCA-*Irgb6*-NheI-fw	CCCCCCGCTAGCATGGCTTGGGCCTCCAGC
PCA-*Irgb6*-NotI-bw	CCCCCCGCGGCCGCTCAAGCTTCCCAGTACTCGGG
PCA-*Irga6*-fw	CCCCCCGCTAGCATGGGTCAGCTGTTCTCTTCACC
PCA-*Irga6*-bw	CCCCCCGCGGCCGCCTAGTTTCTTAAACATATCTCTTTAAGAAGAGTTTTAGCATC
rop39-PCA-fw	CCCCCCGCTAGCCTCCTTGATCGCGTTCTTTCCCGC
rop39-PCA-bw	CCCCCCGCGGCCGCTTAAACAATTGATTCCCGAAGAGGCAAAA
pGEX-*rop39*-fw	CCCCCCGGATCCATGCTCCTTGATCGCGTTCTTTCCCG
pGEX-*rop39*-bw	CCCCCCGCGGCCGCTTAAACAATTGATTCCCGAAGAGGCAAA
pUC-*rop39*-XhoI-fw	CCCCCCCTCGAGGCCGCCACCATGCATCATCATCATCATCATCTGCTGGACCGCTTCTG
pUC-*rop39*-BamHI-bw	CCCCCCGGATCCTTAAACGATGCTCTCACGCAAC
rop39 ko-PCR-fw	ACAACCTCGGCTTTAAACGTGAACC
rop39 ko-PCR-bw	CCACCCGAGGATTATCACAGCCTAT
