# Supplementary material for: ROP39 is an Irgb10-specific parasite effector that modulates acute Toxoplasma gondii virulence
Source: PLoS Pathog. 2023 Jan 5;19(1):e1011003. doi: 10.1371/journal.ppat.1011003 (PMC9848475; doi:10.1371/journal.ppat.1011003)
Supplement: S5 Table — (PDF) [file ppat.1011003.s020.pdf]

S5 Table. Immunoreagents used in this study.

Name	Source
Rabbit polyclonal anti-ROP39 antiserum (11696.20)	This study
Mouse monoclonal anti Irga6 antibody (10E7)	[1]
Rabbit anti Irgb10 antiserum (940/6)	[2]
Mouse anti Irgb6 monoclonal antibody (B34)	[3]
Rabbit anti Irgd antiserum (2078)	[4]
Rat anti GRA7 monoclonal antibody (2.4.21)	[2]
Rabbit anti (pT108)Irga6 antiserum	[5]
Rabbit anti (pT102)Irga6 antiserum	[5]
Mouse anti ROP5 monoclonal antibody	[6]
Alexa Flour 555 donkey anti-mouse	Thermo Fisher Scientific
Alexa Flour 555 donkey anti-rabbit	Thermo Fisher Scientific
Alexa Flour 488 donkey anti-rat	Thermo Fisher Scientific
Peroxidase goat anti-rabbit	Jackson ImmunoResearch
Peroxidase goat anti-mouse	Jackson ImmunoResearch
Peroxidase goat anti-rat	Jackson ImmunoResearch

1. Papic N, Hunn JP, Pawlowski N, Zerrahn J, Howard JC (2008) Inactive and active states of the interferon-inducible resistance GTPase, Irga6, in vivo. The Journal of biological chemistry 283 (46): 32143–32151.
2. Hermanns T, Müller UB, Könen-Waisman S, Howard JC, Steinfeldt T (2016) The Toxoplasma gondii rhoptry protein ROP18 is an Irga6-specific kinase and regulated by the dense granule protein GRA7. Cellular microbiology 18 (2): 244–259.
3. Carlow DA, Teh SJ, Teh HS (1998) Specific antiviral activity demonstrated by TGTP, a member of a new family of interferon-induced GTPases. Journal of immunology (Baltimore, Md. : 1950) 161 (5): 2348–2355.
4. Martens S, Sabel K, Lange R, Uthaiah R, Wolf E et al. (2004) Mechanisms regulating the positioning of mouse p47 resistance GTPases LRG-47 and IIGP1 on cellular membranes: retargeting to plasma membrane induced by phagocytosis. Journal of immunology (Baltimore, Md. : 1950) 173 (4): 2594–2606.
5. Steinfeldt T, Könen-Waisman S, Tong L, Pawlowski N, Lamkemeyer T et al. (2010) Phosphorylation of mouse immunity-related GTPase (IRG) resistance proteins is an evasion strategy for virulent Toxoplasma gondii. PLoS biology 8 (12): e1000576.
6. Leriche MA, Dubremetz JF (1991) Characterization of the protein contents of rhoptries and dense granules of Toxoplasma gondii tachyzoites by subcellular fractionation and monoclonal antibodies. Molecular and biochemical parasitology 45 (2): 249–259. Available: https://pubmed.ncbi.nlm.nih.gov/2038358/.
